# LNS8801 inhibits Acute Myeloid Leukemia by Inducing the Production of Reactive Oxygen Species and Activating the Endoplasmic Reticulum Stress Pathway

**DOI:** 10.1158/2767-9764.CRC-22-0478

**Published:** 2023-08-18

**Authors:** Inyoung Lee, Miriam Doepner, Jillian Weissenrieder, Ariana D. Majer, Sophia Mercado, Angela Estell, Christopher A. Natale, Pamela J. Sung, J. Kevin Foskett, Martin P. Carroll, Todd W. Ridky

**Affiliations:** 1 Department of Dermatology, Perelman School of Medicine, University of Pennsylvania, Philadelphia, Pennsylvania.; 2 Department of Physiology, Perelman School of Medicine, University of Pennsylvania, Philadelphia, Pennsylvania.; 3 Linnaeus Therapeutics, Haddonfield, New Jersey.; 4 Department of Medicine, Roswell Park Comprehensive Cancer Center, Buffalo, New Jersey.; 5 Department of Medicine, Perelman School of Medicine, University of Pennsylvania, Philadelphia, Pennsylvania.

## Abstract

Despite recent therapeutic advances, the 5-year survival rate for adults with acute myeloid leukemia (AML) is poor and standard-of-care chemotherapy is associated with significant toxicity, highlighting the need for new therapeutic approaches. Recent work from our group and others established that the G protein-coupled estrogen receptor (GPER) is tumor suppressive in melanoma and other solid tumors. We performed a preliminary screen of human cancer cell lines from multiple malignancies and found that LNS8801, a synthetic pharmacologic agonist of GPER currently in early phase clinical trials, promoted apoptosis in human AML cells. Using human AML cell lines and primary cells, we show that LNS8801 inhibits human AML in preclinical *in vitro* models, while not affecting normal mononuclear cells. Although GPER is broadly expressed in normal and malignant myeloid cells, this cancer-specific LNS8801-induced inhibition appeared to be independent of GPER signaling. LNS8801 induced AML cell death primarily through a caspase-dependent apoptosis pathway. This was independent of secreted classical death receptor ligands, and instead required induction of reactive oxygen species (ROS) and activation of endoplasmic reticulum (ER) stress response pathways including IRE1α. These studies demonstrate a novel activity of LNS8801 in AML cells and show that targeting ER stress with LNS8801 may be a useful therapeutic approach for AML.

**Significance:**

Previous work demonstrated that LNS8801 inhibits cancer via GPER activation, especially in solid tumors. Here we show that LNS8801 inhibits AML via GPER-independent mechanisms that include ROS induction and ER activation.

## Introduction

Acute myeloid leukemia (AML) is a hematologic malignancy associated with poor differentiation and uncontrolled proliferation of myeloid progenitor cells. For decades, the first-line therapy for AML has remained the “7 + 3” regimen, which consists of a combination of cytotoxic chemotherapeutics anthracycline and cytarabine (1). While this standard of care has a cure rate of approximately 40% in patients younger than 60 years old, efficacy declines sharply in those older than 60 (2–5). In addition, this regimen is poorly tolerated by elderly patients and is associated with higher mortality in patients with comorbidities (6–8). Recurrent AML frequently develops resistance to first-line agents (9), and responses to alternative targeted therapies including decitabine (a DNA-hypomethylating agent) or venetoclax (a Bcl-2 inhibitor) are often modest and short lived (10, 11), highlighting the need for novel therapeutic strategies.

Here we explore the idea that G protein-coupled receptors (GPCR) may be useful therapeutic targets for AML. GPCRs are seven-pass transmembrane receptors typically localized on the cell surface and the endoplasmic reticulum (ER) that regulate diverse cellular processes including metabolism, differentiation, proliferation, and responses to extracellular stimuli (12). They are often misregulated in cancer and play critical roles in the canonical hallmarks of cancer (13, 14). GPCRs are also highly druggable, as nearly 30%–40% of all FDA-approved drugs target are GPCRs (15). Despite their high drugability, only a small number of GPCRs are currently targeted by available cancer therapeutics (16).

Previous reports demonstrated that there are significant GPCR aberrations in AML and that high expression of some GPCRs, including CXCR4, is associated with worse outcomes (17, 18). Imipridones, an emerging class of drugs that targets GPCRs, have shown some efficacy in multiple cancer types including AML (19, 20).

Recently, the G Protein-coupled estrogen receptor (GPER), a GPCR that functions as a nonclassical surface estrogen receptor, has been shown to have tumor suppressive activity in several cancer types (21–25). GPER is distinct from classical nuclear estrogen receptors and has been shown to signal through cAMP and calcium in multiple cellular contexts (26–29). Our group discovered that selective activation of GPER using a synthetic agonist, which does not bind to the classical estrogen receptors, inhibits various cancer types including melanoma and pancreatic ductal adenocarcinoma (30–32). This GPER-specific agonist, G-1, has recently been shown to inhibit hematologic malignancies, such as T-cell lymphoblastic leukemia and AML, through various mechanisms (33, 34). For the first time, we demonstrate that LNS8801, the enantiomerically pure GPER agonist that is currently in early phase clinical trials for melanoma and other advanced malignancies arising in solid organs (31), effectively inhibits AML *in vitro* and in an *in vivo* subcutaneous model. This anti-AML activity is mediated by activation of reactive oxygen species (ROS) and ER stress pathways independent of classical GPER signaling.

## Materials and Methods

### Cell Lines

U937, MOLM14, MV4-11, HL60, and THP1 cell lines were from the lab of Dr. Martin Carroll (Department of Hematology and Oncology, University of Pennsylvania, Philadelphia, PA). A375 line was purchased from ATCC (ATCC CRL-1619). WM46 line was a gift from Dr. Meenhard Herlyn (Wistar Institute, Philadelphia, PA). HPDAC, PANC-1, and MiaPaca-2 were a gift from Dr. Ben Stanger (Department of Medicine, University of Pennsylvania, Philadelphia, PA). All cell lines were authenticated by short tandem repeat profiling performed by the Penn Genomic Analysis Core's DNA Sequencing Laboratory. AML cell lines were cultured in RPMI1640 + GlutaMAX (Gibco, 72400047) supplemented with 5% FBS (Gibco, 26140079) and 1% Penicillin-Streptomycin (Gibco, 15140122) at 37°C with 5% CO_2_. A375, HPDAC, PANC-1, and MiaPaca-2 cells were cultured in DMEM (Gibco, 11995065) with 5% FBS (Gibco, 26140079) and 1% Penicillin-Streptomycin (Gibco, 15140122). WM46 cells were cultured in Tu2% media (400 mL MCDB 153 medium, 100 mL Leibovitz's L-15 medium, 0.5 mL 3.8 ng/mL, 0.35 mL 2.4 mol/L CaCl_2_, 2% FBS, 1% P/S). All cell lines were tested for *Mycoplasma* with the LookOut *Mycoplasma* PCR Detection Kit (Sigma MP0035).

### Reagents

Functional necessity of the different caspases was tested using the pan-caspase inhibitor Q-VD-OPH (Cayman, 15260), caspase-8 specific inhibitor Z-IETD-FMK (BD Biosciences, 550380), and caspase-9 specific inhibitor Z-LEHD-FMK (BD Biosciences, 550381). ER stress inhibitors used in this study include the IRE1α inhibitor Kira6 (Cayman, 19151) and PERK inhibitor GSK2606414 (Cayman, 17376). GPER antagonist G-36 (Cayman, 14397) was used to block CREB phosphorylation. Thapsigargin (Cayman, 10522) was used as a positive control for ER stress experiments. Estradiol (Cayman, 10006315) was used as the endogenous agonist for GPER. N-acetyl-L-Cysteine (NAC; Cayman, 20261) was used as a ROS scavenger.

### WST-8 Assay

Cells were seeded at 3 × 10^3^–2 × 10^4^ cells per 200 μL media in a clear 96-well plate and were incubated with LNS8801 (provided by Linnaeus Therapeutics), LNS8812 (provided by Linnaeus Therapeutics), or G-1 (Cayman, 10008933) for 72 hours at 37 °C with 5% CO_2_. Vehicle (DMSO; Sigma-Aldrich, D8418) concentration was limited to 0.1% for all studies. After incubating cells with appropriate drugs, 10 μL of Cell Counting Kit-8 dye (Dojindo, CK04) were added to each well and absorbance was measured at 450 nm with a Synergy HT plate reader (BioTek). Relative cell number was calculated by the following equation:



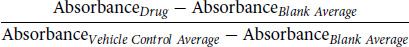



All experiments were done with five to six replicates per condition.

### Cell-cycle Analysis

Cells were plated at a density of 5 × 10^5^ cells/mL one day prior to drug treatment. Upon DMSO control or 250 nmol/L LNS8801 treatment, cells were incubated for 20 hours at 37°C with 5% CO_2_. Cells were then washed with dulbecco's phosphate-buffered saline (DPBS) (Corning 21-031-CV) and permeabilized with chilled 70% ethanol. Cells were stained with FxCycle PI/RNase Staining Solution (Invitrogen, F10797) according to the manufacturer's protocol. Flow cytometry on the cells was performed using a BD LSRII flow cytometer, and flow cytometry graphs were analyzed with FlowJo. All conditions were analyzed in triplicate.

### CRISPR/Cas9 Knockout and Subcloning

MOLM14 cells expressing Cas9 (MOLM14-Cas9) were a gift from the Carroll lab. MOLM14-Cas9 cells were lentivirally transduced with single-guide RNAs targeting the following sequences:

sgGPER3; ATTGAGGTGTTCAACCTGCAC
sgGPER5; CTTCTCCAACAGCTGCCTAAAC

One week after guide RNA transduction, cells were serially diluted so that there was one cell per well in 96-well plates. Single cells were then expanded and used for functional experiments.

### Annexin V/Propidium Iodide Death Assay

AML cell lines were plated at a density of 2.5 × 10^5^ cells/mL and were treated with DMSO or drugs for 72 hours at 37°C with 5% CO_2_. To test the functional necessity of ER stress regulators in LNS8801-induced death, cells were plated at a density of 5 × 10^5^ cells/mL one day prior to drug treatment. Cells were then treated with drugs for 24 hours. After incubation, cells were harvested and washed with DPBS. Cells were then stained with Alexa fluor 488 conjugated Annexin V antibody and propidium iodide (PI) according to the Cell Death Apoptosis Kit manual (Invitrogen, V13245). Flow cytometry on cells was performed using a BD LSRII flow cytometer, and flow cytometry graphs were analyzed with FlowJo. All conditions were analyzed in triplicate.

### Mcl-1 Overexpression

Mcl-1 lentivirus was produced using the pLenti CMV MCL1 Puro construct (Addgene, #140746). U937 cells transduced with the Mcl-1 lentivirus were selected with 1 μg/mL puromycin for 7 days. Mcl-1 overexpression was confirmed via Western blot analysis.

### Colony Formation Assay

AML patient samples and normal bone marrow mononuclear cells were acquired from the Stem Cell and Xenograft Core at the University of Pennsylvania (Philadelphia, PA). All patient samples were collected with written informed consent in accordance with the Declaration of Helsinki and the University of Pennsylvania Institutional Review Board protocol 703185. 2 × 10^4^–4 × 10^4^ cells were plated in 35 mm plates in Human Methylcellulose Enriched Media (R&D Systems, HSC005) with DMSO or 250 nmol/L LNS8801. Plates were incubated for 14 days at 37°C with 5% CO_2_ according to the manufacturer's instruction. Colony number was quantified under the microscope. All conditions were plated in triplicate. Representative images of colonies are provided in the Supplementary Materials and Methods.

### High-speed Spectrofluorimetric Ca++ Measurements

For Ca++ measurements, U937 cells were counted and resuspended to a final concentration of 3 × 10^6^ cells/mL in complete RPMI. A total of 12 × 10^6^ cells were stained with 2 μmol/L Fura2-AM (Thermo Fisher Scientific, F1225) in complete media at 37°C and 5% CO_2_ for 30 minutes in the dark. After staining, cells were pelleted at 1,500 rpm for 3 minutes at 4°C, then the media was aspirated. Cells were washed once with extracellular-like media + Ca++ (ECM + Ca++; 20 mmol/L HEPES, 120 mmol/L NaCl, 5 mmol/L KCl, 1 mmol/L KH_2_PO_4_, 0.2 mmol/L MgCl_2_, 0.1 mmol/L ethylene glycol tetraacetic acid (EGTA), 2 mmol/L CaCl_2_, in MilliQ-H2O, and 1N NaOH to pH = 7.4). Cells were then resuspended to a final volume of 1.5 mL in ECM + Ca++. Fura2-AM fluorescence was excited at 340 and 380 nm with a dual wavelength excitation and emission spectrofluorimeter (Delta Ram, PTI) for a 500-second time course. Compounds of interest (25 nmol/L estradiol and 250 nmol/L LNS8801) were added at 200 seconds. Ionomycin (1 μmol/L), thapsigargin (2 μmol/L), and histamine (100 μmol/L) served as positive controls, whereas DMSO served as a negative vehicle control. Resultant 340/380 ratios were normalized to the equilibrium ratio at 100–200 seconds (R/R0). Experiments were carried out in biological triplicate and are displayed as mean ± SEM.

### Western Blot Analysis

AML cell lines and AML patient samples were washed once with DPBS and lysed with urea lysis buffer (8 mol/L urea, 50 mmol/L NaCl, 50 mmol/L Tris-HCl, pH 8.3, 10 mmol/L dithiothreitol, and 50 mmol/L iodoacetamide). Protein concentration was quantified via Bradford assay, normalized, and reduced with SDS buffer. Protein extract was resolved via SDS gel electrophoresis on 4%–15% Tris/Glycine gels (Bio-Rad, 4561086). Using the Trans-Blot Turbo Transfer System (Bio-Rad), protein was transferred to polyvinylidene difluoride membranes and blocked with 5% BSA in TBS-T. Membranes were then probed with primary antibodies that recognize β-actin (Cell Signaling Technology, #4967, 1:2,000), GPER (Sigma-Aldrich, HPA027052, 1:1,000), Cleaved-caspase 3 (Cell Signaling Technology, #9661, 1:1,000), Cleaved-caspase 8 (Cell Signaling Technology, #9496, 1:1,000), Cleaved-caspase 9 (Cell Signaling Technology, #9505, 1:1,000), XBP-1s (Cell Signaling Technology, #12782, 1:1,000), CHOP (Cell Signaling Technology #2895, 1:1,000), Phospho-CREB (Cell Signaling Technology, #9198, 1:1,000), CREB (Cell Signaling Technologies, #4820, 1:1,000), Mcl-1 (Cell Signaling Technology, #5453, 1:1,000), Bcl-2 (Cell Signaling Technology, #4223, 1:1,000), Bcl-xL (Cell Signaling Technology, #2764, 1:1,000), Bak (Cell Signaling Technology, #3814, 1:1,000), Bax (Cell Signaling Technology, #2772, 1:1,000), Bim (Cell Signaling Technology, #2933, 1:1,000), Puma (Cell Signaling Technology, #12450, 1:1,000), and Noxa (Cell Signaling Technology, #14766, 1:1,000). After membranes were incubated with appropriate primary antibodies, they were incubated with either Anti-mouse IgG, horseradish peroxidase (HRP)-linked Antibody (Cell Signaling Technology, #7076, 1:2,000) or Anti-rabbit IgG, HRP-linked Antibody (Cell Signaling Technology, #7074, 1:2,000) depending on primary antibody source. Proteins were detected using either Clarity Western enhanced chemiluminescence (ECL) Substrate (Bio-Rad, 1705060) or Clarity Max Western ECL Substrate (Bio-Rad, 1705062) and were measured using the ChemiDoc Imaging System (Bio-Rad). Western blots were quantified with ImageJ. All blots were repeated at least three times.

### ROS Detection

For LNS8801 time course experiments with U937 and MOLM14, cells were treated with 250 nmol/L LNS8801 for 2 and 4 hours. Following LNS8801 treatment, cells were harvested and washed with DPBS. After washing, cells were resuspended in PBS with 2% FBS containing 10 μmol/L 2′,7′-Dichlorofluorescein diacetate (DCF). Cells were then incubated at 37°C for 30 minutes in the dark. Finally, cells were washed with PBS and resuspended in PBS with 2% FBS for FACS analysis. Flow cytometry on cells were performed using a BD LSRII flow cytometer, and flow cytometry graphs were analyzed with FlowJo. To test the necessity of ROS induction for LNS8801-induced death, U937 and MOLM14 cells were pretreated with 4 mmol/L NAC for 1 hour. After pretreatment, cells were treated with 250 nmol/L LNS8801 for 8 hours. Cells were prepared and analyzed for ROS detection as outlined above.

### Xenograft Model

Subcutaneous tumors were grafted by injecting 10 × 10^6^ MOLM14 cells in 50% Matrigel (Corning, 356234) into the left flank of male nonobese, diabetic, severe combined immunodeficiency γ_chain−/−_ mice (NSG, provided by the Stem Cell and Xenograft Core at the University of Pennsylvania, Philadelphia, PA). LNS8801 was dissolved in a vehicle formulation of 85% sesame oil, 5% ethanol, and 10% DMSO or LNS8801 in DMSO. Once tumor size reached 150–300 mm^3^ mice were treated with either vehicle control or 10 mg/kg LNS8801 daily by oral gavage. Tumors were measured with a caliper. Volume was calculated using the formula *L* × *W*^2^ × 0.52, where *L* is the longest dimension and *W* is the perpendicular dimension. Mice were euthanized when the tumor volume exceeded 1,000 mm^3^ or due to secondary endpoints including severe ulceration, death, and any other condition that falls within the Institutional Animal Care and Use Committee (IACUC) guidelines for Rodent Tumor and Cancer Models at the University of Pennsylvania (Philadelphia, PA). For the systemic model, luciferase-expressing MOLM14 or MV4-11 cells were injected into tail veins of NSG mice after busulfan conditioning. Tumor burden was assessed 2 or 4 days after tail vein injection via bioluminescent imaging on an IVIS Spectrum after mice were injected with d-luciferin (PerkinElmer). Upon confirmation of engraftment, mice with MV4-11 cells were treated daily with either vehicle or 10 mg/kg LNS8801 orally. Mice with MOLM14 cells were treated daily with either vehicle or 100 mg/kg LNS8801 orally. Mice that met the endpoint conditions outlined in the IACUC guidelines for Rodent Tumor and Cancer Models at the University of Pennsylvania (Philadelphia, PA) were euthanized. All rodent experiments for this study have been approved by IACUC protocol 803381.

### Statistical Analysis

All statistical analyses were performed using GraphPad Prism 8 (GraphPad Software). No statistical methods were used to predetermine sample size. Details of each statistical test used are included in the figure legends.

### Data Availability

The data generated in this study are available upon request from the corresponding author.

## Results

### LNS8801 Inhibits Human AML *in vitro*

**FIGURE 1:**
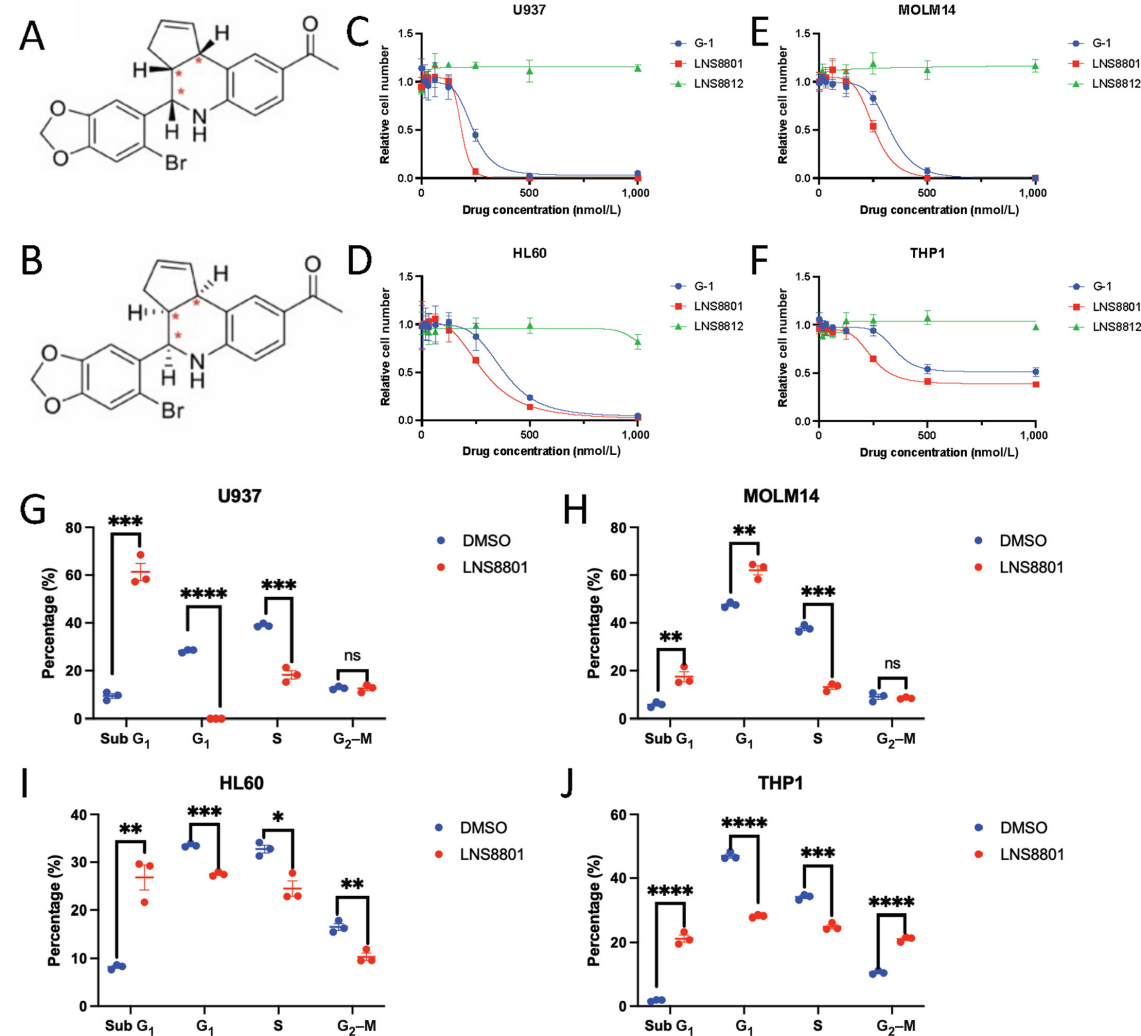
GPER agonist LNS8801 inhibits AML *in vitro*. Structure of LNS8801 (**A**) and LNS8812 (**B**). Concentration**–**response curves done with G-1, LNS8801, and LNS8812 in U937 (**C**), MOLM14 (**D**), HL60 (**E**), and THP1 (**F**) AML cell lines. Cells were incubated with the respective drugs for 72 hours. Relative cell count was determined using WST-8 dye. Six replicates were used per condition. Cell-cycle analysis done with PI staining in U937 (**G**), MOLM14 (**H**), HL60 (**I**), and THP1 (**J**) treated. Cells were treated with 250 nmol/L LNS8801 for 20 hours. Three replicates were used per condition. *P* values were calculated via pairwise *t* tests for G–J (*, *P* ≤ 0.05; **, *P* ≤ 0.01; ***, *P* ≤ 0.001; ****, *P* ≤ 0.0001).

All prior studies in the literature utilizing a GPER-specific agonist use the synthetic compound G-1 (35). While G-1 is highly selective for GPER over nuclear estrogen receptors, it is a racemic mixture of two enantiomers. As new racemic mixtures are generally not advanced for human use, G-1 was separated into LNS8801 and LNS8812, which do not interconvert (Fig. 1A and B) (31). All of G-1′s antitumor activity appears to reside in LNS8801, which is the compound currently in human trials.

We tested LNS8801 across a panel of cancer cell lines and found that LNS8801 inhibited growth in multiple cancer types but seemed to promote apoptosis in AML cells (Supplementary Fig. S1). GPER protein is expressed in AML cell lines and primary AML samples isolated from patients, as well as normal mononuclear cells (Supplementary Fig. S2A and S2B). To test whether LNS8801 activates classical GPER signaling pathways in AML, we treated primary AML cells with LNS8801 and/or the GPER-specific antagonist G-36 (36) and looked for changes in cAMP-responsive element binding protein (CREB) phosphorylation, as CREB phosphorylation is a well-established downstream readout of GPER activation (32, 37, 38). As expected, we found that LNS8801 induced CREB phosphorylation and that the effect was blocked by G-36, suggesting that LNS8801 engages and signals through GPER in primary AML cells (Supplementary Fig. S2C).

In a panel of commonly used human AML cell lines, we confirmed that the racemic agonist, G-1, and its active enantiomer, LNS8801, reduced relative cell numbers, while the inactive enantiomer LNS8812 failed to do so (Fig. 1C–F). Consistent with the known heterogeneity in AML drug responses, we noted a range of LNS8801 sensitivity among the four lines (Supplementary Table S1), with U937 being the most sensitive and THP1 being the most resistant. LNS8801 sensitivity did not seem to correlate with mutational status including FLT3 mutation. Similarly, clinical outcomes do not seem to correlate with GPER expression according to BEAT-AML dataset (Supplementary Fig. S3).

Cell-cycle analysis showed that LNS8801 induced an appreciable decrease in the number of cells in S-phase and a significant increase in sub-G_1_-phase across all AML cell lines (Fig. 1G–J). The loss of cells in S-phase suggests that LNS8801 inhibits proliferation, and the increase in cells in sub-G_1_-phase indicates that LNS8801 compromises cell viability. Together, these data show that LNS8801 effectively inhibits AML *in vitro*.

### LNS8801 Inhibits AML Colony Formation

**FIGURE 2:**
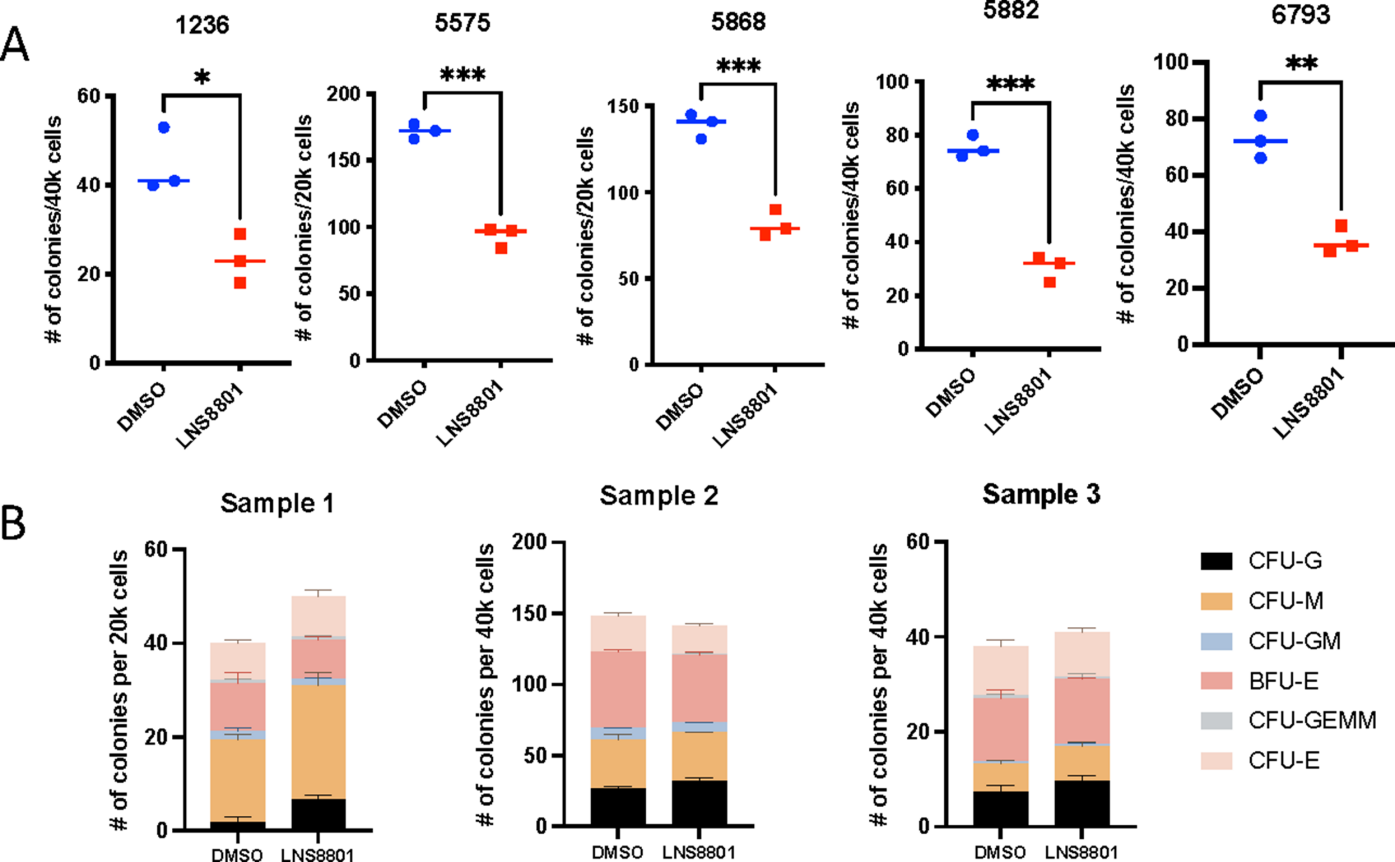
LNS8801 inhibits AML colony formation. **A,** Colony formation assay of primary AML cells in either DMSO or 250 nmol/L LNS8801. Three replicates were used per condition. **B,** Colony formation of normal mononuclear cells in either DMSO or 250 nmol/L LNS8801. Three replicates were used per condition. CFU: Colony forming units. BFU: Blast forming units. CFU-G: CFU-granulocytes. CFU-M: CFU-monocytes. CFU-GM: CFU-granulocyte/monocyte. BFU-E: BFU-Erythrocytes. CFU-GEMM: CFU-granulocyte/erythrocyte/monocyte/megakaryocyte. CFU-E: CFU-erythrocyte *P* values were calculated using *t* tests (*, *P* ≤ 0.05; **, *P* ≤ 0.01; ***, *P* ≤ 0.001).

To test whether the anti-AML activity observed in the established AML lines extended to human primary AML cells isolated directly from patients, we performed colony formation assays using AML patient samples and normal bone marrow mononuclear cells, with or without LNS8801. While LNS8801 significantly reduced the number of colonies in AML patient samples, it did not decrease colony forming capacity in normal mononuclear cells (Fig. 2A and B; Supplementary Fig. S4). Colony number and morphology with the normal mononuclear cells were unaffected by LNS8801, which is consistent with the lack of LNS8801 toxicity observed in humans (Supplementary Fig. S4; ref. 31). Characteristics of the primary AML cells are outlined in Supplementary Table S2.

### LNS8801-induced AML Inhibition is Independent of Canonical GPER Signaling

**FIGURE 3:**
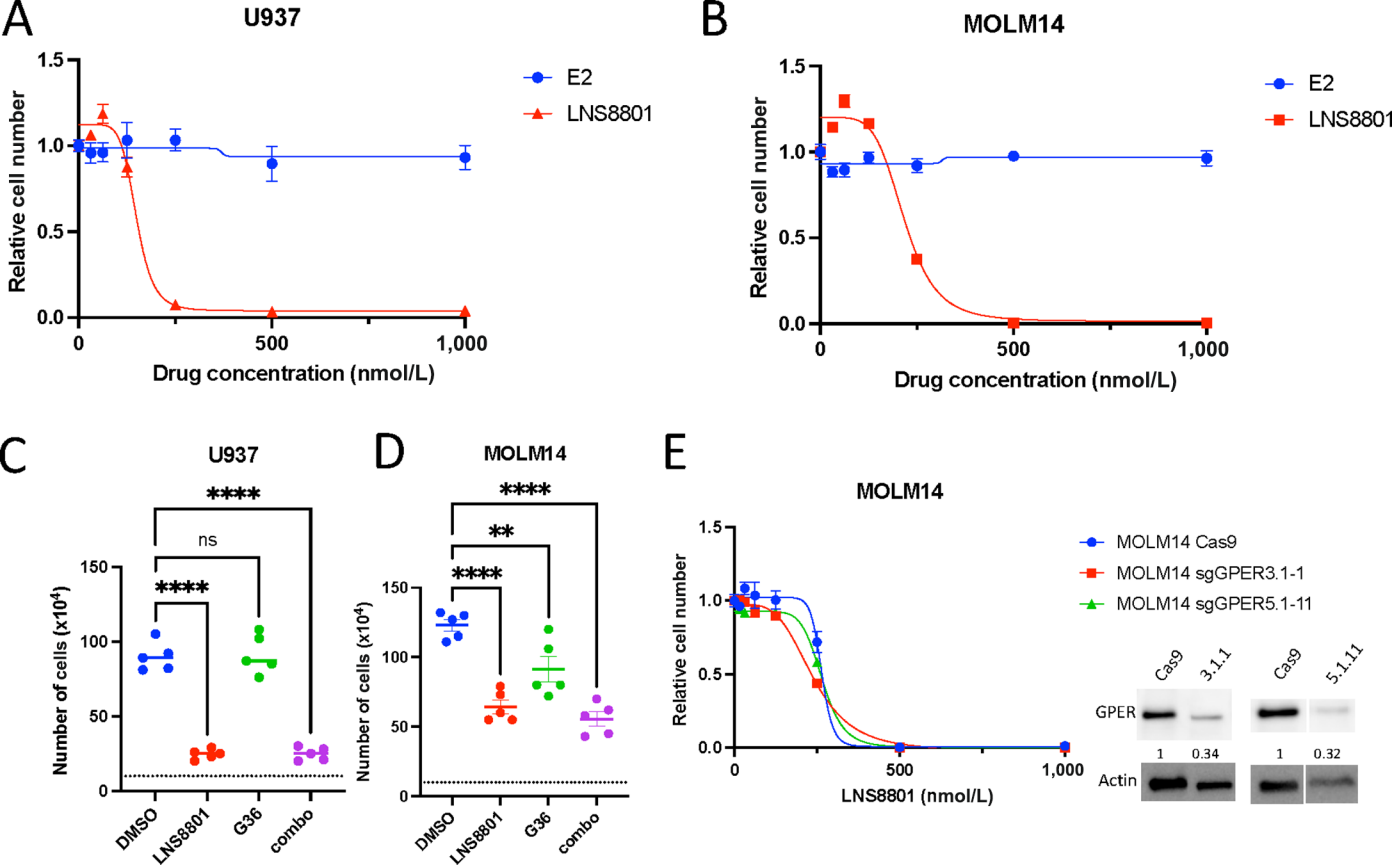
LNS8801 inhibits AML independently of canonical GPER signaling. Concentration–response curves done with LNS8801 and estradiol (E2) in U937 (**A**) and MOLM14 (**B**). Cells were incubated for 72 hours. Proliferation assay performed with 125 nmol/L LNS8801 and/or 1 μmol/L G36 in U937 (**C**) and MOLM14 (**D**). Five replicates were used per condition. Cells were incubated for 72 hours. **E,** LNS8801 concentration–response curve in MOLM14 GPER knockdown cells. Representative Western blots show GPER expression in knockdown cells. Full blot is available in Supplementary Fig. S4. One-way ANOVA with multiple comparisons was used to calculate *P* values for C and D (*, *P* ≤ 0.05; **, *P* ≤ 0.01; ***, *P* ≤ 0.001; ****, *P* ≤ 0.0001).

We next tested whether the anti-AML effect of LNS8801 was mediated through GPER and subsequent signaling. To test whether GPER activation leads to AML inhibition, we performed proliferation assays using the endogenous GPER ligand estradiol (E2). Unlike LNS8801, E2 did not inhibit the proliferation of U937 or MOLM14 cells (Fig. 3A and B). Furthermore, we tested the necessity of GPER signaling for LNS8801-induced AML inhibition by using the GPER-specific antagonist G-36 and found that G-36 did not prevent the antiproliferative effect of LNS8801 (Fig. 3C and D). Finally, we took a genetic approach and partially depleted GPER in MOLM14 using CRISPR-Cas9. After subcloning GPER-depleted cells, we established clonal lines with approximately 70% depletion of the GPER protein (Supplementary Fig. S5A). Consistent with previous data, the sensitivity of GPER-depleted cells to LNS8801 was similar to the sensitivity of parental cells (Fig. 3E).

GPER canonically activates G_s_ signaling, which increases the intracellular cAMP concentration (39, 40). To understand whether GPER activation induces cAMP in AML cells, we measured phosphorylation of CREB, which is a downstream readout of cAMP induction. While we observed an increase in CREB phosphorylation in some AML cell lines and primary AML cells, this did not correlate with LNS8801 sensitivity (Supplementary Fig. S6A and S6B). The CREB phosphorylation in AML cell lines with LNS8801 was less pronounced that what was observed in primary AML. Despite this variability in CREB phosphorylation, LNS8801 inhibited proliferation in all AML cell lines tested. To further examine whether cAMP signaling contributes to LNS8801-induced inhibition, we treated U937 cells with cAMP analogs and found that none sufficiently inhibited AML cell line growth (Supplementary Fig. S7A), suggesting that cAMP is not a significant contributor of the LNS8801-induced inhibition in AML.

In other settings, GPER activation leads to increased cytosolic [Ca^2+^] (40). However, calcium was not affected by LNS8801 nor by E2 in AML, although calcium changes were readily detected after exposure to the thapsigargin, histamine, and ionomycin positive controls (Supplementary Fig. S7B). This result suggests that LNS8801-induced AML inhibition is also independent of a [Ca^2+^] flux.

### LNS8801 Induces Caspase-dependent Apoptosis in AML

**FIGURE 4:**
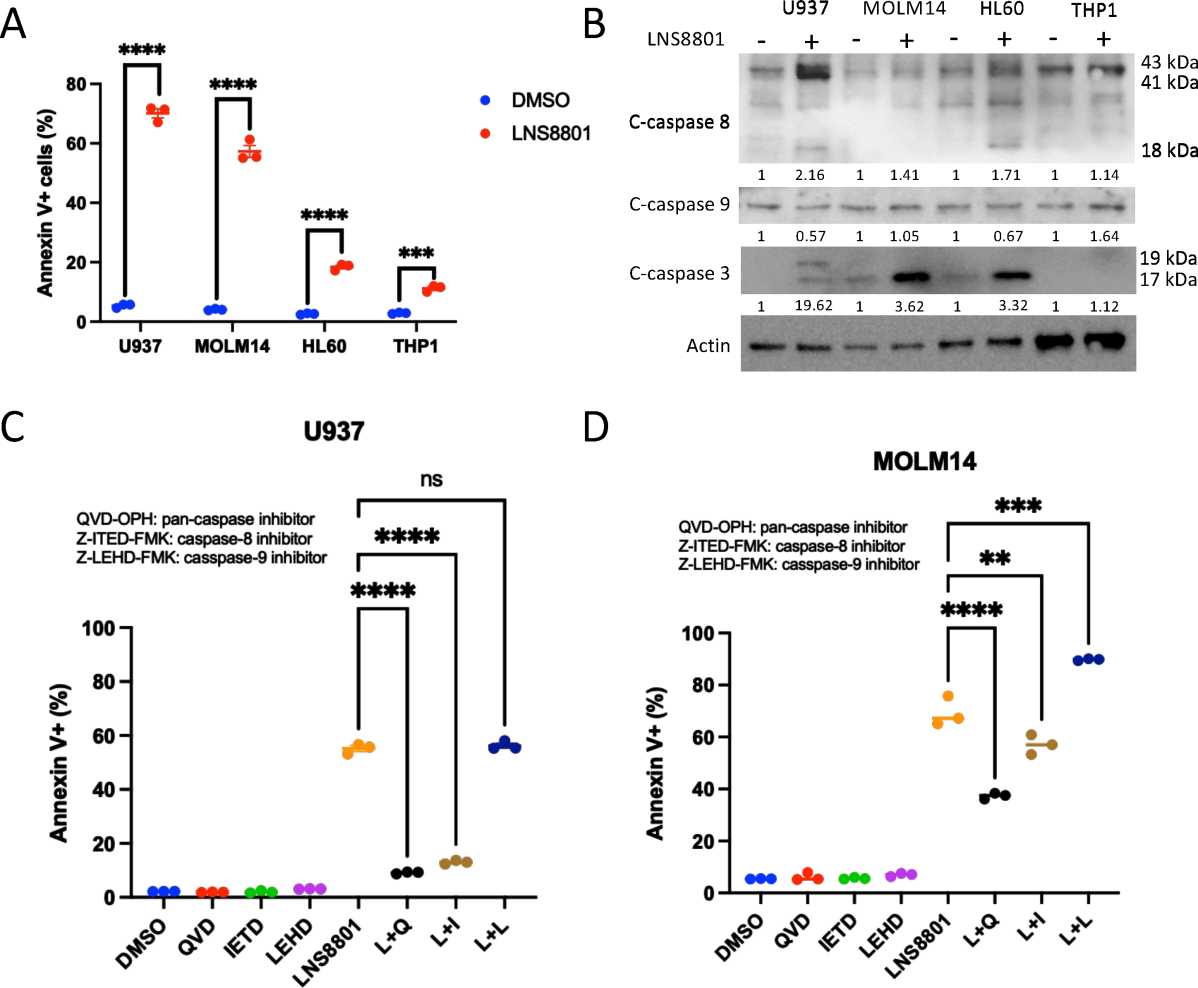
Caspase cleavage mediates LNS8801-induced death. **A,** Annexin V+ flow cytometry analysis of human AML cell lines treated with 250 nmol/L LNS8801 for 72 hours. Three replicates were used per condition. **B,** Western blot analysis of AML cells treated with 250 nmol/L LNS8801 for 24 hours. Western blot quantification was done by normalizing bands to actin bands. Annexin V+ flow cytometry analysis of U937 (**C**) and MOLM14 (**D**) cells treated with LNS8801 and specific caspase inhibitors. A total of 250 nmol/L LNS8801, 10 μmol/L QVD-OPH (QVD), 50 μmol/L (U937), and 20 μmol/L (MOLM14) for Z-IETD-FMK (IETD), and 10 μmol/L Z-LEHD-FMK (LEHD) were used. L+Q indicates LNS8801 and QVD-OPH, L+I indicates LNS8801+Z-IETD-FMK, and L+L indicates LNS8801 and Z-LEHD-FMK. Three replicates were used per condition. Statistical analysis was done via pairwise *t* tests for A and one-way ANOVA with multiple comparisons was used for C and D (*, *P* ≤ 0.05; **, *P* ≤ 0.01; ***, *P* ≤ 0.001; ****, *P* ≤ 0.0001).

As LNS8801 increased the proportion of cells in the sub-G_1_-phase (Fig. 1G–J), we hypothesized that LNS8801 induced apoptosis. Consistent with this, LNS8801 treatment increased Annexin V positivity across all cell lines, suggesting that cells died by apoptosis (Fig. 4A). Apoptosis is classically trigged by either the intrinsic or extrinsic apoptotic pathways. The intrinsic apoptotic pathway is regulated by the antiapoptotic Bcl-2 family proteins including Bcl-2, Bcl-xL, and Mcl-1, and the proapoptotic proteins including Bak and Bax. Extreme and extended intracellular stress alters Bcl-2 family protein levels to promote mitochondria pore formation and *cytochrome c* release, which initiates downstream death signaling pathways (41). In AML, the intrinsic apoptotic pathway is commonly inhibited, but it can sometimes be effectively reengaged in human patients via pharmacologic Bcl-2 inhibition with venetoclax. This led us to question whether the intrinsic apoptotic pathway was responsible for LNS8801-induced cell death. While we did not observe appreciable LNS8801-induced changes in proapoptotic proteins (namely, Bak, Bax, and Bim), we did see consistent depletion of the antiapoptotic protein Mcl-1 across all cell lines (Supplementary Fig. S8). Other relevant antiapoptotic proteins in AML, such as Bcl-2 and Bcl-xL, were grossly unchanged upon LNS8801 treatment (Supplementary Fig. S8).

Mcl-1 protein is often overexpressed in various cancers and is necessary for AML survival (42–45). To test whether Mcl-1 loss was sufficient to recapitulate the apoptosis we observed following LNS8801 exposure, we pharmacologically inhibited Mcl-1 with S63845 (46) in our panel of AML cell lines. Surprisingly, the LNS8801 highly sensitive line U937 was completely resistant to S63845, while the other lines were exquisitely sensitive to the drug (Supplementary Fig. S9A). We next tested whether restoring Mcl-1 protein expression in the face of LNS8801 exposure prevented LNS8801-induced cell death and found that Mcl-1 overexpression did not prevent LNS8801-induced apoptosis (Supplementary Fig. S9B and S9C). Together, these data suggest that LNS8801-induced Mcl-1 loss is not a major determinant of LNS8801-induced cell death in AML.

To further clarify whether LNS8801-induced AML cell death was mediated by the extrinsic or intrinsic apoptosis pathways, we next determined protein levels of cleaved caspases 3, 8, and 9 following treatment with LNS8801. While we observed upregulation of cleaved caspase-8 (extrinsic pathway) and cleaved caspase-3 (downstream effector of both the extrinsic and the intrinsic pathway) in most cell lines, cleaved caspase-9 (intrinsic pathway) was not notably altered by LNS8801 treatment with the exception of the THP1 cell line (Fig. 4B). This indicates that caspases are involved in LNS8801-induced death but that the specific subtype involvement in AML may be context dependent.

Next, to test whether caspase cleavage was a necessary mediator of LNS8801-induced death, we used QVD-O-PH (pan-caspase inhibitor), Z-IETD-FMK (caspase-8 inhibitor), and Z-LEHD-FMK (caspase-9 inhibitor). As predicted, the pan-caspase and caspase-8 inhibitor significantly reduced Annexin V positivity in LNS8801-treated U937 cells, while the caspase-9 inhibitor had no effect (Fig. 4C). In MOLM14 cells, the pan-caspase and caspase-8 inhibitor had a more modest effect, indicating that there is heterogeneity in caspase involvement in LNS8801-induced death response (Fig. 4D). These data are consistent with the idea that LNS8801-induced cell death is mediated by activated caspases, including the caspase specific to the extrinsic pathway.

Extrinsic apoptosis is classically triggered by secreted death ligands. Therefore, we questioned whether LNS8801 induces extrinsic cell death by increasing production of these classical death ligands including TNFα, FasL, or TRAIL. We found that there was a robust increase in TNFα expression upon LNS8801 treatment, while FasL and TRAIL were grossly unchanged (Supplementary Fig. S10A). To test whether the increased TNFα was responsible for LNS8801-induced death, we pretreated AML cells with an anti-TNFR1 blocking antibody and treated cells with LNS8801. While anti-TNFR1 antibody pretreatment blocked death induced by exogenous TNFα, it did not block death induced by LNS8801, suggesting that the LNS8801-induced TNFα increase is not sufficient to kill cells (Supplementary Fig. S10B).

To test whether different secreted factors activated the extrinsic apoptosis pathway following LNS8801 exposure, we treated AML cells with LNS8801 for 24 hours and collected conditioned media. We then removed LNS8801 from that media via dialysis through a 10,000 molecular weight cut-off (MWCO) membrane, which should retain death ligand proteins. This dialyzed conditioned media did not induce significant cell death, indicating that the “extrinsic” apoptosis observed following LNS8801 is likely initiated by cell intrinsic factors rather than a classical autocrine/paracrine death ligand (Supplementary Fig. S10C).

### LNS8801-induced ROS Production Inhibits AML

**FIGURE 5:**
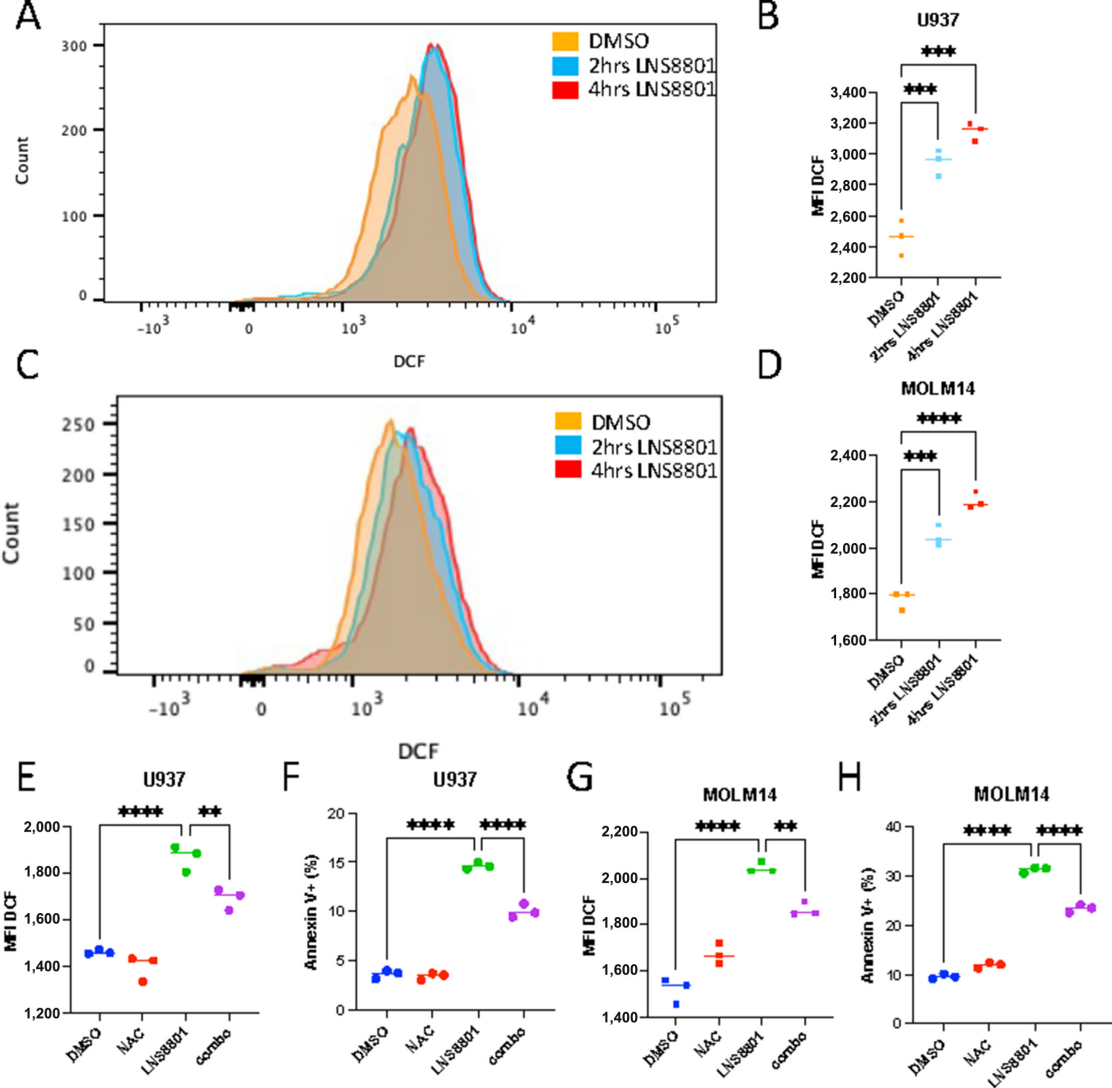
ROS induction mediates LNS8801-induced cell death. Representative DCF histogram (**A**) and summary graph (**B**) of median fluorescence intensity (MFI) of U937 cells treated with 250 nmol/L LNS8801. Representative DCF histogram (**C**) and summary graph (**D**) of MFI of MOLM14 cells treated with 250 nmol/L LNS8801. Three replicates were analyzed per condition. MFI DCF (**E**) and annexin V+ flow cytometry analysis (**F**) of U937 cells treated with 4 mmol/L NAC and/or 250 nmol/L LNS8801. MFI DCF (**G**) and annexin V+ flow cytometry analysis (**H**) of MOLM14 cells treated with 4 mmol/L NAC and/or 250 nmol/L LNS8801. Cells were pretreated with NAC or vehicle for 1 hour, followed by an 8-hour incubation with LNS8801 or vehicle for MFI DCF measurements. Cells were pretreated with 4 mmol/L NAC or vehicle for 1 hour, followed by 24-hour treatment of 250 nmol/L LNS8801 or vehicle for annexin V+ measurements. Statistical analysis was done via one-way ANOVA with multiple comparisons was used for B, D, and E–H (*, *P* ≤ 0.05; **, *P* ≤ 0.01; ***, *P* ≤ 0.001; ****, *P* ≤ 0.0001).

Recent reports show that that G-1 induces ROS in other cell settings (33, 47). ROS may cause DNA damage that induces apoptosis, and is a potent inhibitor of AML via various mechanisms (48, 49). ROS levels were measured using DCF in U937 and MOLM14 cells after 2 and 4 hours of treatment with LNS8801. LNS8801 significantly increased the levels of ROS in both cell lines, while the inactive enantiomer LNS8812 did not induce ROS (Fig. 5A–D; Supplementary Fig. S11). We then tested whether LNS8801-induced ROS upregulation is necessary for AML death. The ROS scavenger NAC blunted LNS8801-induced ROS production as well as the associated cell death (Fig. 5E–H). While we tried to test whether higher concentration of NAC further reversed the effect of LNS8801, we were unable to do so as NAC itself was toxic at higher concentrations, which is consistent with observations from other groups (50, 51). Together, these data show that LNS8801 leads to increased production of ROS, which induces apoptosis in AML cells.

### LNS8801 Induces Apoptosis Through the ER Stress Pathway

**FIGURE 6:**
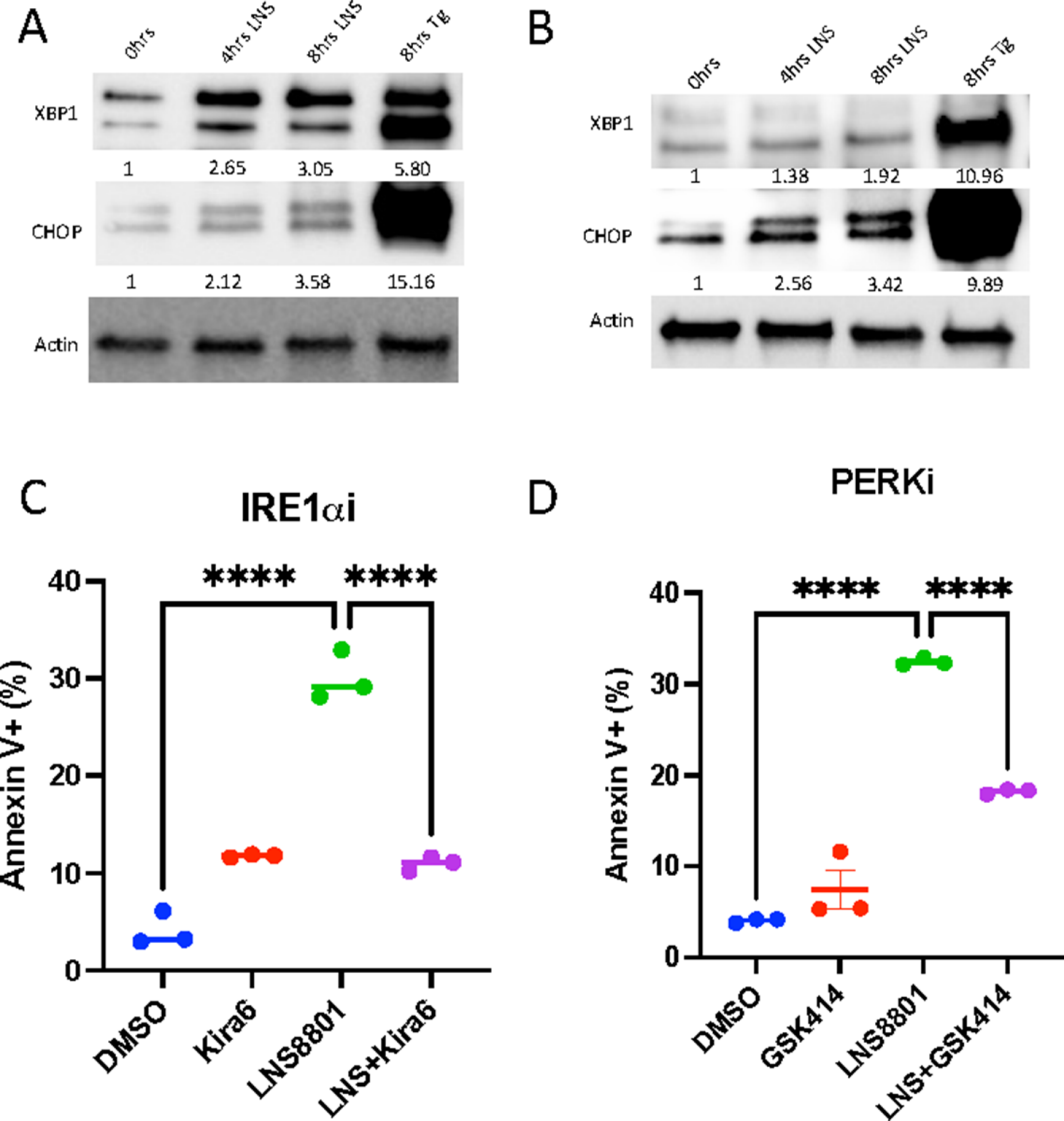
LNS8801 induces apoptosis in AML cells via ER stress response. Western blot analysis of ER stress proteins in response to LNS8801 in U937 (**A**) and MOLM14 (**B**) cells. A total of 250 nmol/L LNS8801 (LNS) and 50 nmol/L thapsigargin (Tg) were used for this experiment. Shown Western blots are representative of at least three experiments. XBP1 and CHOP quantification was done by normalizing to actin bands. Annexin V flow cytometry analysis done with 250 nmol/L LNS8801 and/or (**C**) 1 μmol/L IREα inhibitor Kira6 and 5 μmol/L PERK inhibitor GSK414 for 24 hours. Three replicates were used per condition. *P* values in **C** and **D** were calculated via one-way ANOVA with multiple comparisons (*, *P* ≤ 0.05; **, *P* ≤ 0.01; ***, *P* ≤ 0.001; ****, *P* ≤ 0.0001).

ROS-induced cell death is often associated with ER stress (52–54), and G-1 induces ER stress pathway in some solid tumor models (24, 55). Therefore, we questioned whether LNS8801 induced apoptosis in AML via the ER stress pathway. The main ER stress sensors that govern apoptosis are IRE1α and PERK (56). Upon activation of IRE1α and PERK, downstream effectors including XBP1 and CHOP are upregulated, which then drive apoptosis (57, 58). To test whether LNS8801 induced activation of these ER stress responses in AML, we determined levels of ER stress proteins and noted increases in both XBP1 and CHOP (Fig. 6A and B). Unlike LNS8801, E2 did not induce any noticeable increase in XBP1 or CHOP expression (Supplementary Fig. S12A and S12B).

To test whether these ER stress responses are a necessary for LNS8801-induced cell death, we used ER stress inhibitors in combination with LNS8801. Strikingly, we found that the IRE1α inhibitor, Kira6, completely blocked LNS8801-induced apoptosis in U937 cells (Fig. 6C). The PERK inhibitor, GSK2606414 (GSK414), modestly blunted LNS8801-induced apoptosis in U937 cells (Fig. 6D). Kira6 blocked the cleavage of both caspase-8 and caspase-3, while GSK414 was not able to do so (Supplementary Fig. S13). Together, these data suggest that IRE1α-XBP1 and, to some extent, PERK-CHOP are responsible for driving LNS8801-induced cell death in AML.

### LNS8801 Inhibits AML in Subcutaneous *In Vivo* Model but not Systemic Model

**FIGURE 7:**
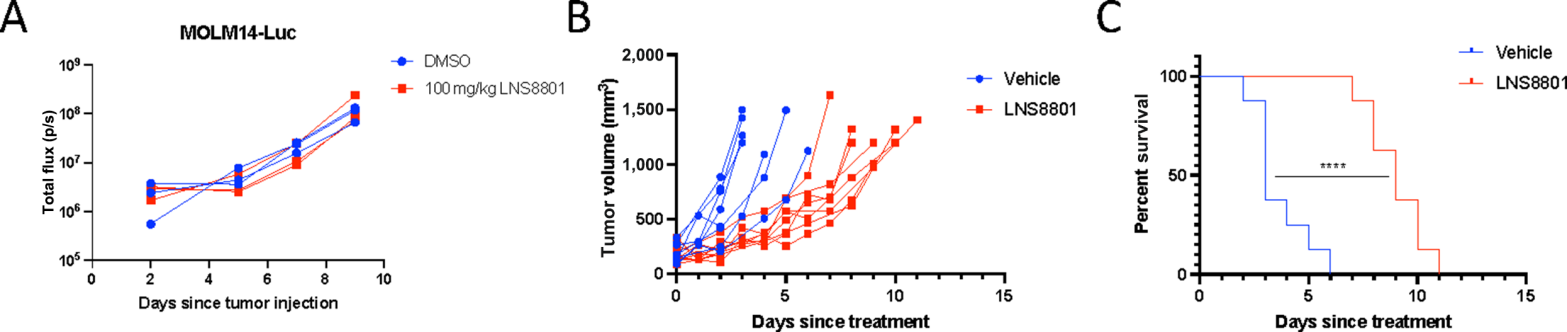
LNS8801 inhibits AML in subcutaneous *in vivo* model but not systemic model. **A,** Systemic *in vivo* experiment using luciferase-expressing MOLM14 cells (MOLM14-Luc). Male NSG mice were used for the experiment. Mice were treated with vehicle or LNS8801 daily through oral gavage. Three animals were used per condition. **B,** Subcutaneous tumor volume of MOLM14 in NSG mice treated orally with vehicle or 10 mg/kg LNS8801 daily. Graph shows summary of two independent experiments. Conditions for each experiment were identical. Four mice were used per group per experiment, totaling 8 mice per condition. **C,** Survival curve of NSG mice with subcutaneous MOLM14 tumors. Statistical analysis for the survival curve was done via log-rank (Mantel–Cox) test and Grehan–Breslow–Wilcoxon test (****, *P* ≤ 0.0001).

We next tested whether LNS8801 inhibited AML in preclinical *in vivo* models. Initially, NSG mice were injected with luciferase-expressing MOLM14 cells (MOLM14-Luc) to test the efficacy of LNS8801 in a clinically relevant setting. Mice harboring MOLM14-Luc xenografts were treated once daily with orally delivered LNS8801 (100 mg/kg). This dosage is approximately 20–100 times higher than what has been used in other preclinical models (30, 32, 59). To our surprise, LNS8801 did not inhibit AML in the systemic model (Fig. 7A). This observation was consistent in another AML cell line, MV4-11, that is sensitive to LNS8801 *in vitro* (Supplementary Fig. S14A and S14B). To understand whether LNS8801 inhibits AML in different *in vivo* contexts, we engrafted MOLM14 cells to NSG mice subcutaneously. Xenografted mice were treated once daily with orally delivered LNS8801 (mice were treated with 10 mg/kg LNS8801, which is approximately 2–10 times the dosage of what has been used in other preclinical models (30, 32, 59)) once tumors reached 150–250 mm^3^ in size. LNS8801 significantly inhibited MOLM14 tumor growth and extended median survival from 3 to 9 days (Fig. 7B and C).

## Discussion

Here, we show that LNS8801 promotes apoptosis in AML through upregulation of ROS, activation of ER stress, and downstream initiation of caspase-dependent apoptosis pathways. Consistent with the lack of toxicity observed in ongoing clinical studies, LNS8801 did not affect normal mononuclear cells.

While these data suggest that LNS8801 inhibits AML through caspase activation, the death appears independent of classical secreted death ligands and changes in Bcl-2 family proteins. Rather, AML death is dependent on ROS and ER stress responses. GPER signaling canonically induces an increase in both intracellular cAMP and calcium. However, our data suggest that the effects of LNS8801 on apoptosis in AML do not depend on these classical G-protein signaling pathways.

As GPCR signaling can often vary depending on cell context (49), it is possible that LNS8801 inhibits AML via nonclassical GPER signaling. Upon activation, GPCRs release the βγ G-protein subunit that has known signaling properties. It has been shown that the βγ subunit coupled to GPER increases MAPK signaling in different cellular contexts (50), indicating that there are multiple pathways outside of the canonical GPER signaling pathway that may mediate the effect of LNS8801. This is consistent with recent data from other groups demonstrating that G-1 inhibits some AML lines through a GPER-p38 signaling axis (34). In addition, GPCR kinases may play a role, as it is becoming evident that they have signaling capacity independent of GPCRs that could potentially explain some of nonclassical GPER signaling (60).

We attempted to deplete GPER protein in AML cells using both short hairpin RNA and CRISPR-Cas9 approaches. While we observed reductions in GPER protein, these were not complete nor durable, as GPER protein quickly returned to baseline levels, making definitive functional experiments challenging. It is likely that acute GPER loss is not tolerated in AML cell lines. To circumvent technical limitation, we used the GPER-specific antagonist G-36, which was unable to block LNS8801 induced AML cell death. In addition, reduction in GPER protein using genetic approaches did not alter sensitivity to LNS8801. This is consistent with findings in uveal melanoma, where LNS8801 also induces cell death *in vitro* independent of GPER (59). More complete and durable GPER depletion will be required to understand the effect of GPER-dependent signaling in AML and definitively exclude a role for GPER in LNS8801-induced AML death. Nonetheless, based on the data presented here, it appears that LNS8801 promotes AML cell death independent of classical GPER signaling.

It should be noted that while most of the AML cell lines treated with LNS8801 upregulated cleaved caspase-8, there was only a modest increase in cleaved caspase-8 and a more notable increase in cleaved caspase-9 in the (relatively) LNS8801-resistant line THP1. Also, while the caspase-8 inhibitor effectively blunted LNS8801-induced apoptosis in U937 cells, its effect was less pronounced in MOLM14 cells. This suggests that LNS8801 can promote apoptosis in AML cells through multiple mechanisms that are not limited to caspase-8 cleavage. Consistent with this, G-1 induced pyroptosis in addition to apoptosis in AML cell lines OCI-AML2 and KG1a (34). These alternative mechanisms warrant further investigation, as AML is a highly heterogeneous disease and the mechanisms described in this work may not capture all of the AML complexity.

Ren and colleagues demonstrated that depletion of Mcl-1 is a major mediator for G-1/venetoclax combination-induced cell death in AML through GPER-p38 signaling, but did not investigate whether Mcl-1 depletion is necessary for G-1–induced death (34). To test whether Mcl-1 depletion plays a role in LNS8801-induced death, we overexpressed Mcl-1 in AML cells so that Mcl-1 protein is maintained when treated with LNS8801 and found that the rescue of Mcl-1 did not prevent cell death. Taken together, these data suggest that LNS8801 may be therapeutically useful in the context of venetoclax-resistant AML, because one of the major mechanisms of venetoclax resistance is driven by Mcl-1 upregulation.

While LNS8801 clearly inhibits AML in *in vitro* contexts, the *in vivo* efficacy is unclear, as LNS8801 inhibited AML only in the subcutaneous model and not the systemic model. Likely explanations for this discrepancy include pharmacokinetic differences between tissues and compensatory mechanisms provided by the bone marrow niche. LNS8801 is highly fat soluble, mostly protein bound, and metabolized fairly quickly in mice, so it is possible that AML cells in systemic models treated once daily with LNS8801 are not sufficiently exposed to bioavailable compound in the blood (61). Furthermore, because the subcutaneous tissue is high in fat (62), LNS8801 may concentrate in the subcutaneous space and thereby contribute to increased AML sensitivity to LNS8801 in the subcutaneous versus the circulating *in vivo* models. It is well known that the bone marrow niche provides compensatory survival and proliferation advantages to leukemic cells through cytokines and endothelial cell support, indicating that AML cells in the systemic model may be rescued by tumor microenvironmental factors (63, 64).

LNS8801 is well tolerated in humans and appears efficacious in some patients with solid tumors (65); however, the efficacy of LNS8801 in people with hematologic malignancies has not yet been tested. While LNS8801 did not inhibit AML in the systemic model, it may still be effective in patients because the half-life is significantly longer in humans; however, a higher concentration of LNS8801 may be required for AML compared with solid tumors. Furthermore, combination therapy with LNS8801 may prove effective to overcome potential compensatory mechanisms associated with the bone marrow niche. Importantly, of the five primary AML cell samples used in our study, all were sensitive to LNS8801, including two samples (5575 and 5868) isolated from a secondary leukemic and a patient with relapsed leukemic. These types of leukemia are mostly resistant to conventional chemotherapy. If LNS8801 proves to be an effective agent in patients with AML, future trials testing the efficacy of LNS8801 in patients with refractory AML may be warranted.

## Supplementary Material

Supplementary Table S1 Supplementary Table S1

Supplementary Figure S1 Supplementary Figure S1. AML cells are particularly sensitive to LNS8801 (A-F) Proliferation assay performed with (A) A375, (B) WM46, (C) Mel290, (D) PANC-1, (E) MiaPaca-2, and (F) U937 cells. Dotted line represents the number of cells seeded. Cells were treated with 250nM LNS8801 for 72 hours. 5 replicates were used per condition per cell line. P values were calculated via pairwise t-tests (*p=<0.05, **p=<0.01, ***p=<0.001, ****p=<0.0001).

Supplementary Table S2 Supplementary Table S2

Supplementary Figure S2 Supplementary Figure S2. Human AML cell lines, primary MNC, and AML cells express GPER (A and B) Western blot of GPER expression in (A) AML cell lines, (B) normal mononuclear cells (MNC), and primary human AML samples. (C) Western blot of a primary AML sample treated with 250nM LNS8801 and/or 1uM G36. Cells were pretreated with G36 for 1 hour and treated with LNS8801 for 1 hour after pretreatment.

Supplementary Figure S3 Supplementary Figure S3. GPER expression grossly does not correlate with AML outcome and mutation status (A) GPER expression stratified by ELN2017. (B) GPER expression stratified by FLT3-ITD mutation status. (C) GPER expression stratified by different AML fusion proteins. All data were acquired from the BEAT-AML dataset. P-values were calculated via one-way ANOVA multiple comparisons (*p=<0.05, **p=<0.01, ***p=<0.001, ****p=<0.0001).

Supplementary Figure S4 Supplementary Figure S4. Colony morphology of human AML cells and normal mononuclear cells (A and B) Brightfield image of colony formation of primary AML cells treated with (A) DMSO and (B) 250nM LNS8801. (C-H) Brightfield image of normal mononuclear cell colony morphologies.

Supplementary Figure S5 Supplementary Figure S5. GPER knockdown in MOLM14 Western blot of GPER knockdown clones of MOLM14 cells.

Supplementary Figure S6 Supplementary Figure S6. LNS8801 induces heterogeneous phosphorylation of CREB in AML (A) Western blot showing AML cell lines treated with either vehicle DMSO or 250nM LNS8801. Cells were treated for 30 minutes. pCREB quantification was normalized to CREB and Actin. (B) Western blots of primary AML samples treated with different doses of LNS8801. Cells were incubated for 1 hour. pCREB quantification was normalized to CREB and Actin.

Supplementary Figure S7 Supplementary Figure S7. LNS8801-induced AML inhibition is independent of classical GPER signaling (A) Annexin V+ flow cytometry analysis of U937 cells treated with 250nM LNS8801, 10uM Forskolin, 100uM EPAC-specific cAMP (8-pCPT-2'-O-Me-cAMP), and 100uM PKA-specific cAMP (N6-benzoyl-cAMP). Cells were incubated for 24 hours. 3 replicates per condition were used. (B) Detection of intracellular calcium upon drug treatment via a spectrofluorimeter assay. Fura2-AM dye was used for detection. 25nM E2, 250nM LNS8801, 1uM Ionomycin, 2uM thapsigargin, and 100uM histamine were used. 3 replicates were used per condition.

Supplementary Figure S8 Supplementary Figure S8. LNS8801 depletes Mcl-1 in AML cells while not altering other Bcl-2 family proteins (A and B) (A) Western blot of pro-apoptotic proteins from U937 cells treated with 250nM LNS8801 as a time course. (B) Western blot of anti-apoptotic proteins treated with 250nM LNS8801 for 24 hours.

Supplementary Figure S9 Supplementary Figure S9. Mcl-1 is not responsible for LNS8801-induced death in AML (A) Proliferation assay dose curve of AML cells treated with the Mcl-1 specific inhibitor S63845. Cells were treated with drug for 72 hours. 6 replicates were used per dose. (B) Western blot of U937 cells overexpressing Mcl-1, treated with 250nM LNS8801 for 24 hours. (C) Annexin V flow analysis of U937 cells overexpressing Mcl-1. Cells were treated for 72 hours. 3 replicates were used per condition. Two-way ANOVA was used for statistical analysis for Annexin V+ flow cytometry comparing wild-type and Mcl-1 overexpressing U937 cells.

Supplementary Figure S10 Supplementary Figure S10. LNS8801 induces cell death independently of the classical secreted death ligands (A) Time course western blot of U937 cells treated with 250nM LNS8801. (B) Annexin V staining flow analysis of U937 cells treated with either 20ng/mL TNF⍺, 10ug/mL TNFR1 antibody, or 250nM LNS8801. Cells were incubated for 72 hours. 3 replicates were used per condition. (C) Annexin V staining flow analysis of U937 cells treated with LNS8801-treated U937 conditioned media. Cells were incubated in conditioned media for 72 hours. 3 replicates were used per condition. One-way ANOVA was used for statistical analysis (*p=<0.05, **p=<0.01, ***p=<0.001, ****p=<0.0001).

Supplementary Figure S11 (A and C) Representative DCF histograms of (A) U937 and (C) MOLM14 cells treated with 250nM LNS8812 or 250nM LNS8801 at indicated time points. (B and D) Summary graph of median fluorescence intensity (MFI) of (B) U937 and (D) MOLM14 cells treated with 250nM LNS8812 or 250nM LNS8801. 3 replicates were analyzed per condition. Statistical analysis was done via one-way ANOVA with multiple comparisons was used for panels B and D (*p=<0.05, **p=<0.01, ***p=<0.001, ****p=<0.0001).

Supplementary Figure S12 (A and B) Western blots of (A) U937 and (B) MOLM14 cells treated with 25nM estradiol (E2) or 50nM thapsigargin (Tg) for indicated time. Western blot quantification was done by normalizing each band to its respective actin band.

Supplementary Figure S13 Supplementary Figure S13. IRE1⍺ inhibitor blocks LNS8801-induced caspase cleavage Western blot of U937 cells treated with 250nM LNS8801 and/or 1uM IRE1⍺ inhibitor Kira6 or 5uM PERK inhibitor GSK414 for 24 hours. Western blot quantification was done by normalizing each band to the respective actin band.

Supplementary Figure S14 LNS8801 does not inhibit AML in systemic in vivo model (A) Proliferation assay of luciferase-expressing MV4-11 (MV4-11-Luc) cells. Cells were incubated for 72 hours and were counted with trypan blue dye. 5 replicates were used for experiment. P-values were calculated via pairwise t-tests (*p=<0.05, **p=<0.01, ***p=<0.001, ****p=<0.0001). (B) Systemic in vivo experiment done with MV4-11-Luc. Male NSG mice were used for the experiment. Mice were treated with vehicle or LNS8801 daily through oral gavage. 3 animals were used per condition.
