# Supplementary material for: LNS8801 inhibits Acute Myeloid Leukemia by Inducing the Production of Reactive Oxygen Species and Activating the Endoplasmic Reticulum Stress Pathway
Source: Cancer Res Commun. 2023 Aug 18;3(8):1594–606. doi: 10.1158/2767-9764.CRC-22-0478 (PMC10438922; doi:10.1158/2767-9764.CRC-22-0478)
Supplement: Supplementary Table S1 [file crc-22-0478-s01.pptx]

## Slide 1
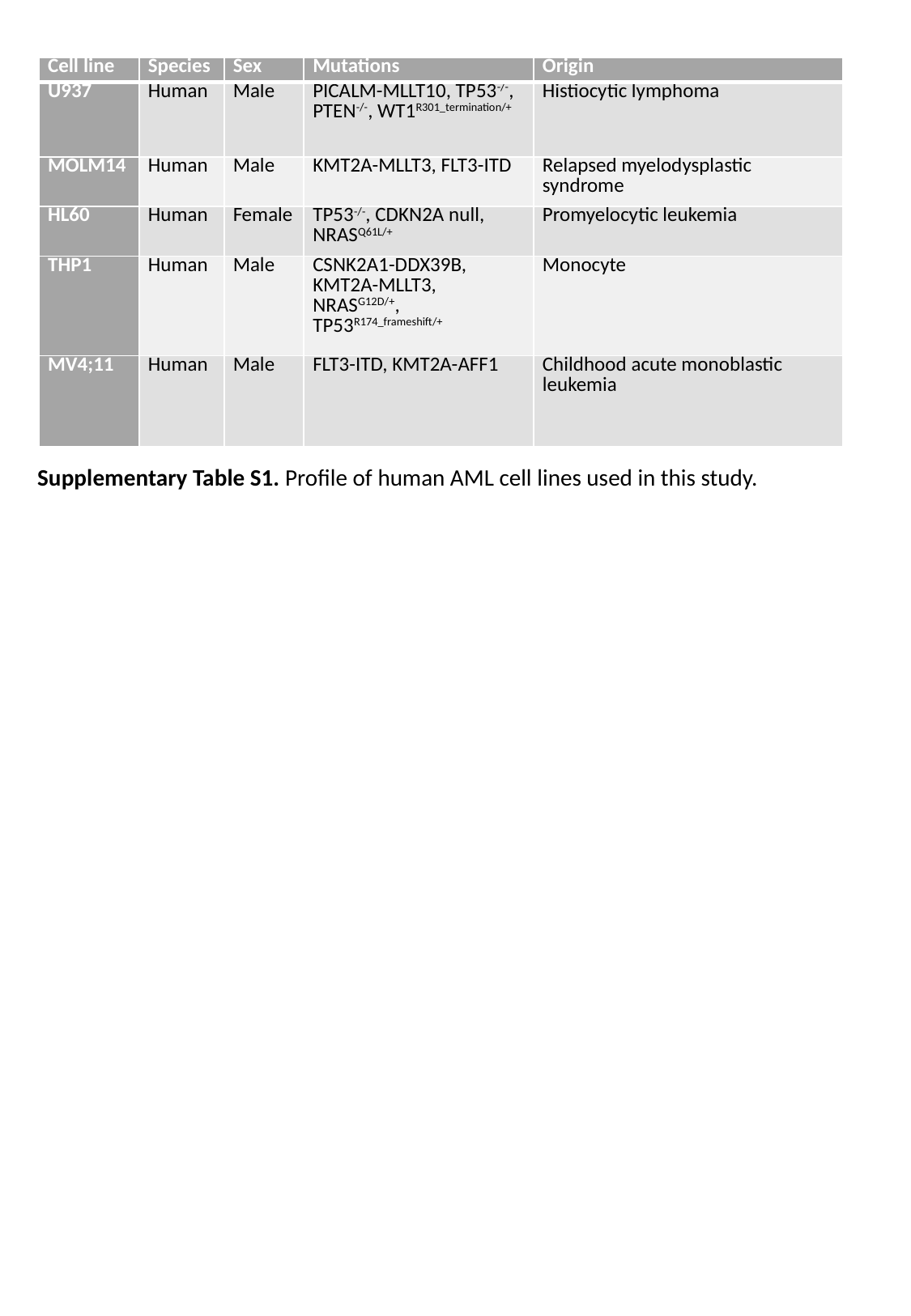

| Cell line | Species | Sex | Mutations | Origin |
| --- | --- | --- | --- | --- |
| U937 | Human | Male | PICALM-MLLT10, TP53-/-, PTEN-/-, WT1R301\_termination/+ | Histiocytic lymphoma |
| MOLM14 | Human | Male | KMT2A-MLLT3, FLT3-ITD | Relapsed myelodysplastic syndrome |
| HL60 | Human | Female | TP53-/-, CDKN2A null, NRASQ61L/+ | Promyelocytic leukemia |
| THP1 | Human | Male | CSNK2A1-DDX39B, KMT2A-MLLT3, NRASG12D/+, TP53R174\_frameshift/+ | Monocyte |
| MV4;11 | Human | Male | FLT3-ITD, KMT2A-AFF1 | Childhood acute monoblastic leukemia |
Supplementary Table S1. Profile of human AML cell lines used in this study.
