# Supplementary figures and images for: LNS8801 inhibits Acute Myeloid Leukemia by Inducing the Production of Reactive Oxygen Species and Activating the Endoplasmic Reticulum Stress Pathway
Source: Cancer Res Commun. 2023 Aug 18;3(8):1594–606. doi: 10.1158/2767-9764.CRC-22-0478 (PMC10438922; doi:10.1158/2767-9764.CRC-22-0478)

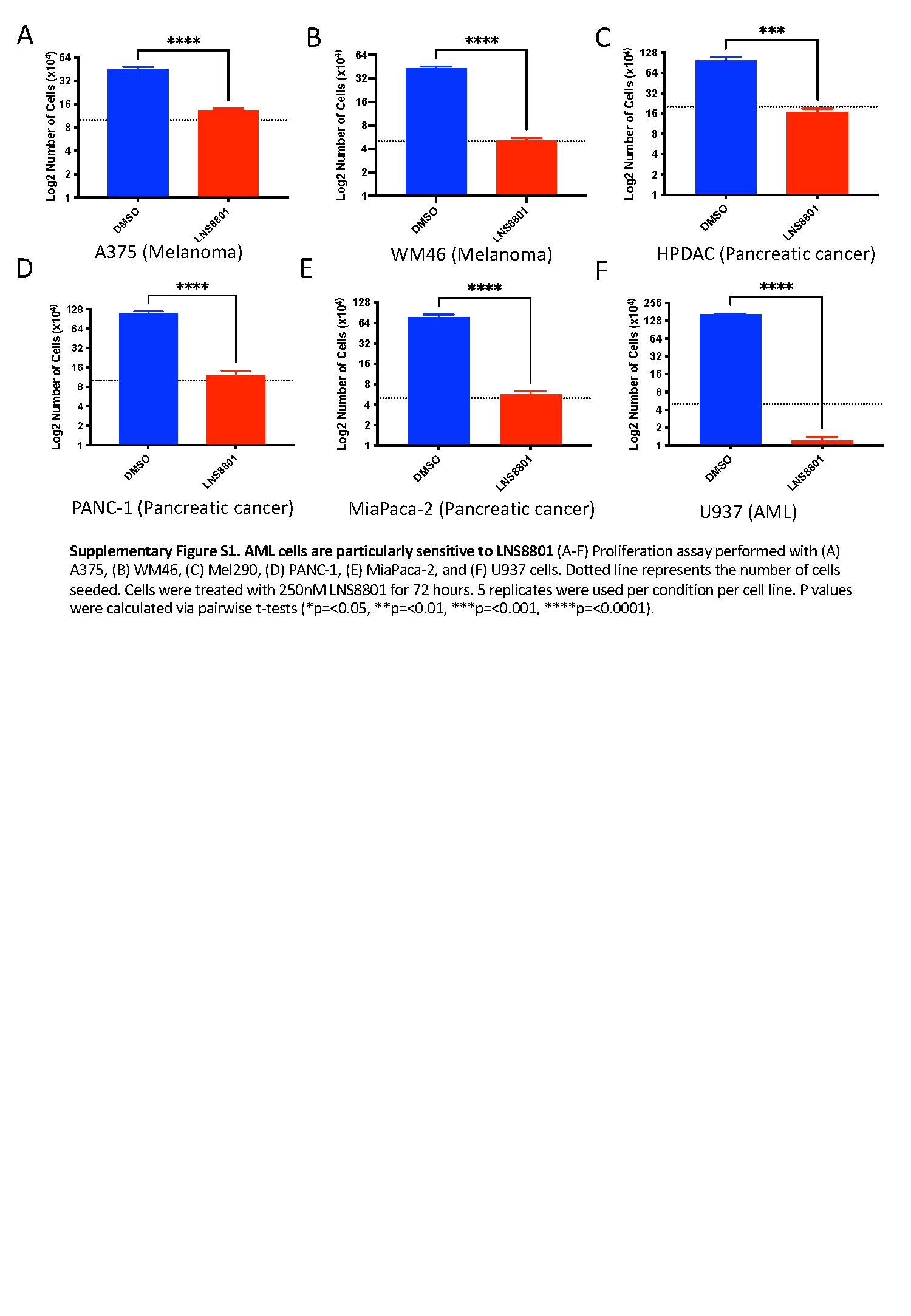

Supplement: Supplementary Figure S1 — AML cells are particularly sensitive to LNS8801 (A-F) Proliferation assay performed with (A) A375, (B) WM46, (C) Mel290, (D) PANC-1, (E) MiaPaca-2, and (F) U937 cells. Dotted line represents the number of cells seeded. Cells were treated with 250nM LNS8801 for 72 hours. 5 replicates were used per condition per cell line. P values were calculated via pairwise t-tests (*p=<0.05, **p=<0.01, ***p=<0.001, ****p=<0.0001). [file crc-22-0478-s02.png]

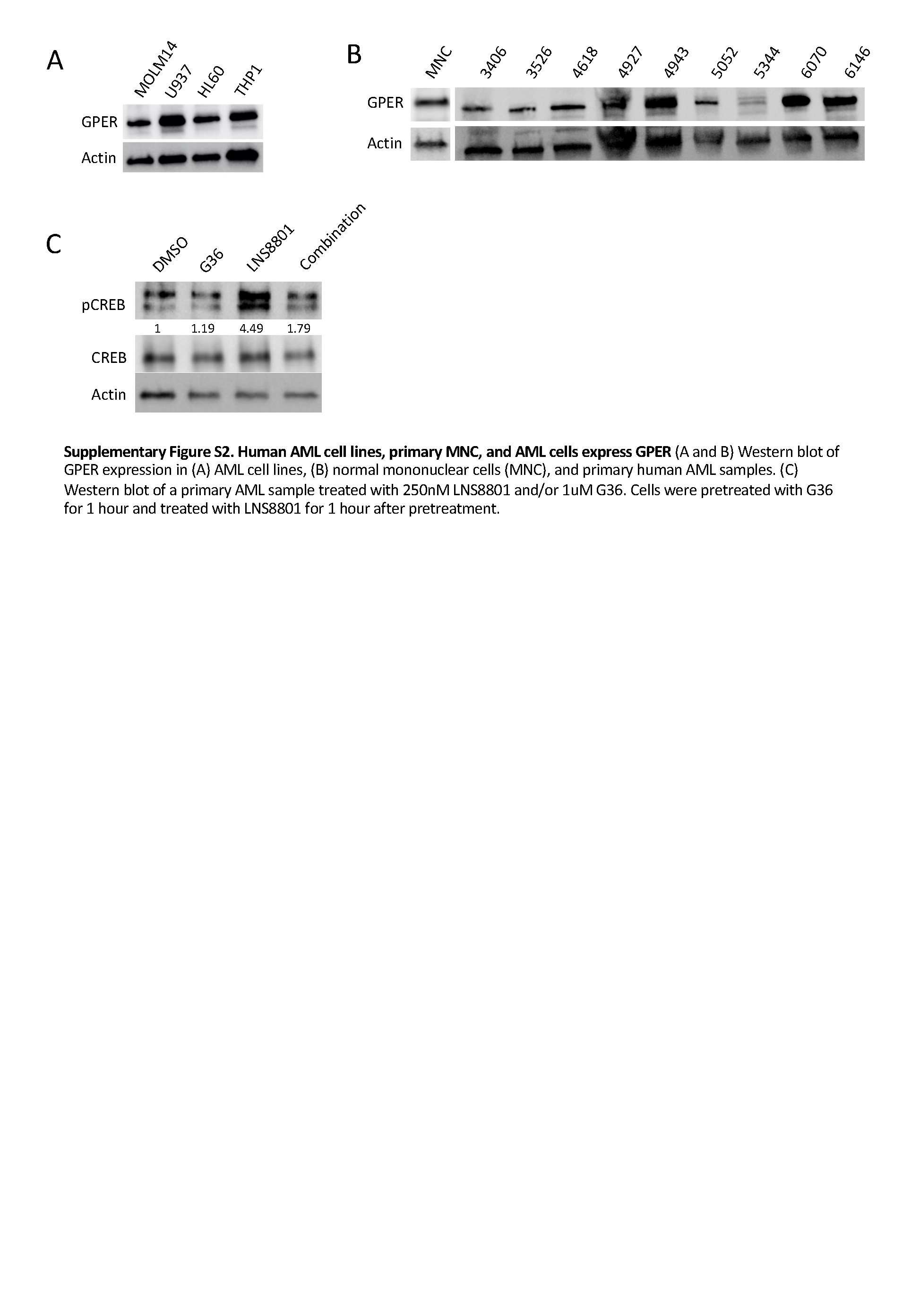

Supplement: Supplementary Figure S2 — Human AML cell lines, primary MNC, and AML cells express GPER (A and B) Western blot of GPER expression in (A) AML cell lines, (B) normal mononuclear cells (MNC), and primary human AML samples. (C) Western blot of a primary AML sample treated with 250nM LNS8801 and/or 1uM G36. Cells were pretreated with G36 for 1 hour and treated with LNS8801 for 1 hour after pretreatment. [file crc-22-0478-s04.png]

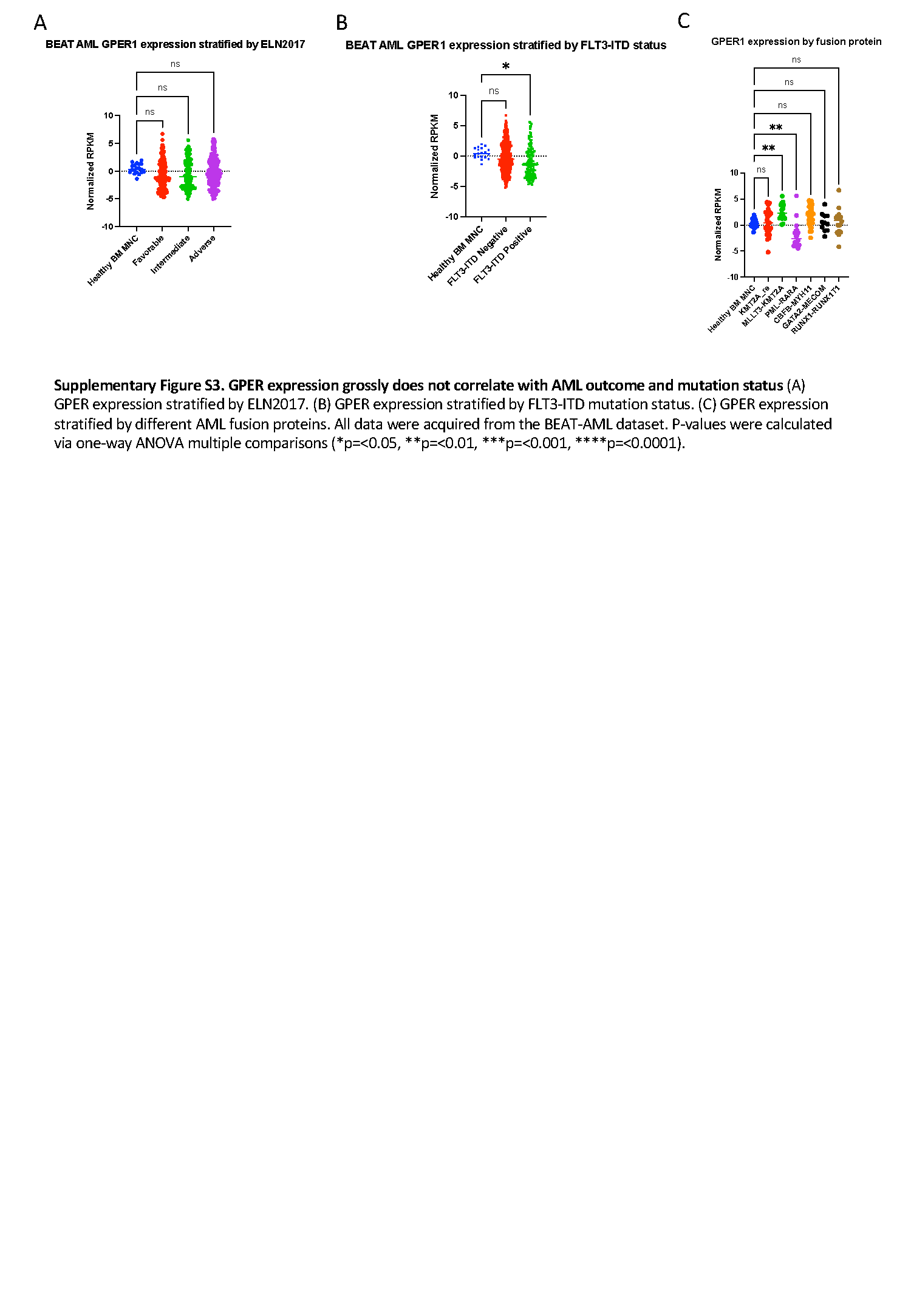

Supplement: Supplementary Figure S3 — GPER expression grossly does not correlate with AML outcome and mutation status (A) GPER expression stratified by ELN2017. (B) GPER expression stratified by FLT3-ITD mutation status. (C) GPER expression stratified by different AML fusion proteins. All data were acquired from the BEAT-AML dataset. P-values were calculated via one-way ANOVA multiple comparisons (*p=<0.05, **p=<0.01, ***p=<0.001, ****p=<0.0001). [file crc-22-0478-s05.png]

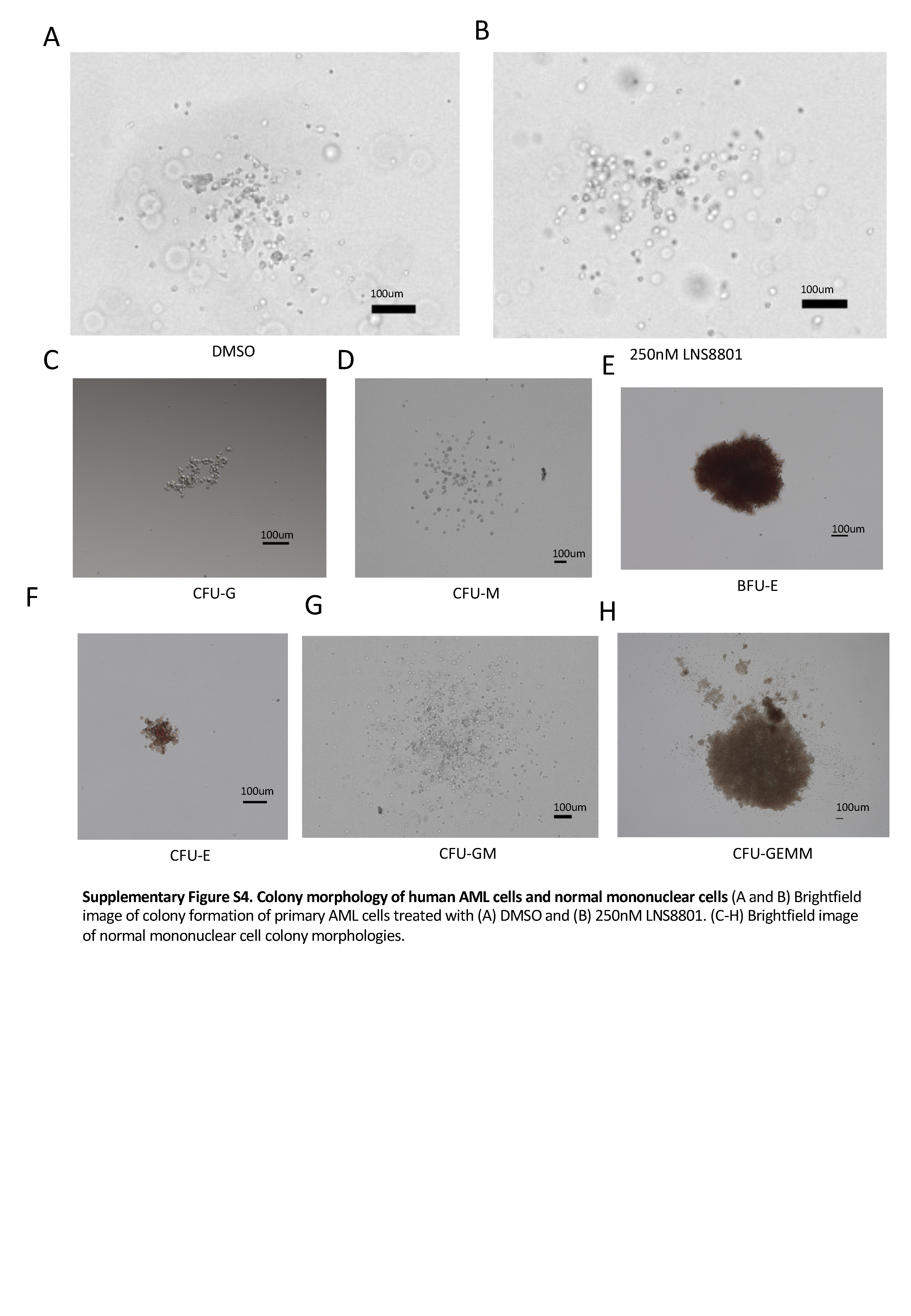

Supplement: Supplementary Figure S4 — Colony morphology of human AML cells and normal mononuclear cells (A and B) Brightfield image of colony formation of primary AML cells treated with (A) DMSO and (B) 250nM LNS8801. (C-H) Brightfield image of normal mononuclear cell colony morphologies. [file crc-22-0478-s06.png]

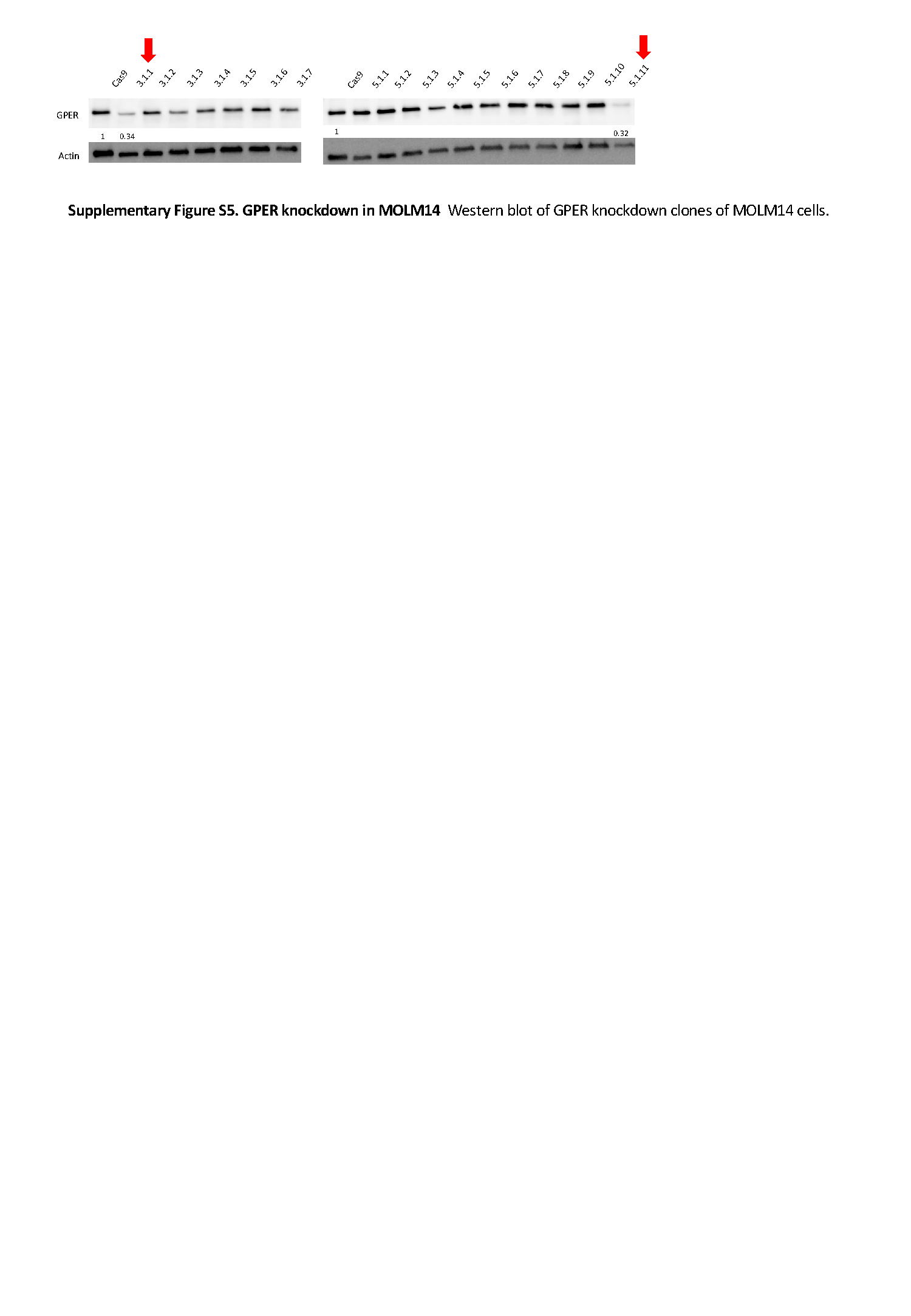

Supplement: Supplementary Figure S5 — GPER knockdown in MOLM14 Western blot of GPER knockdown clones of MOLM14 cells. [file crc-22-0478-s07.png]

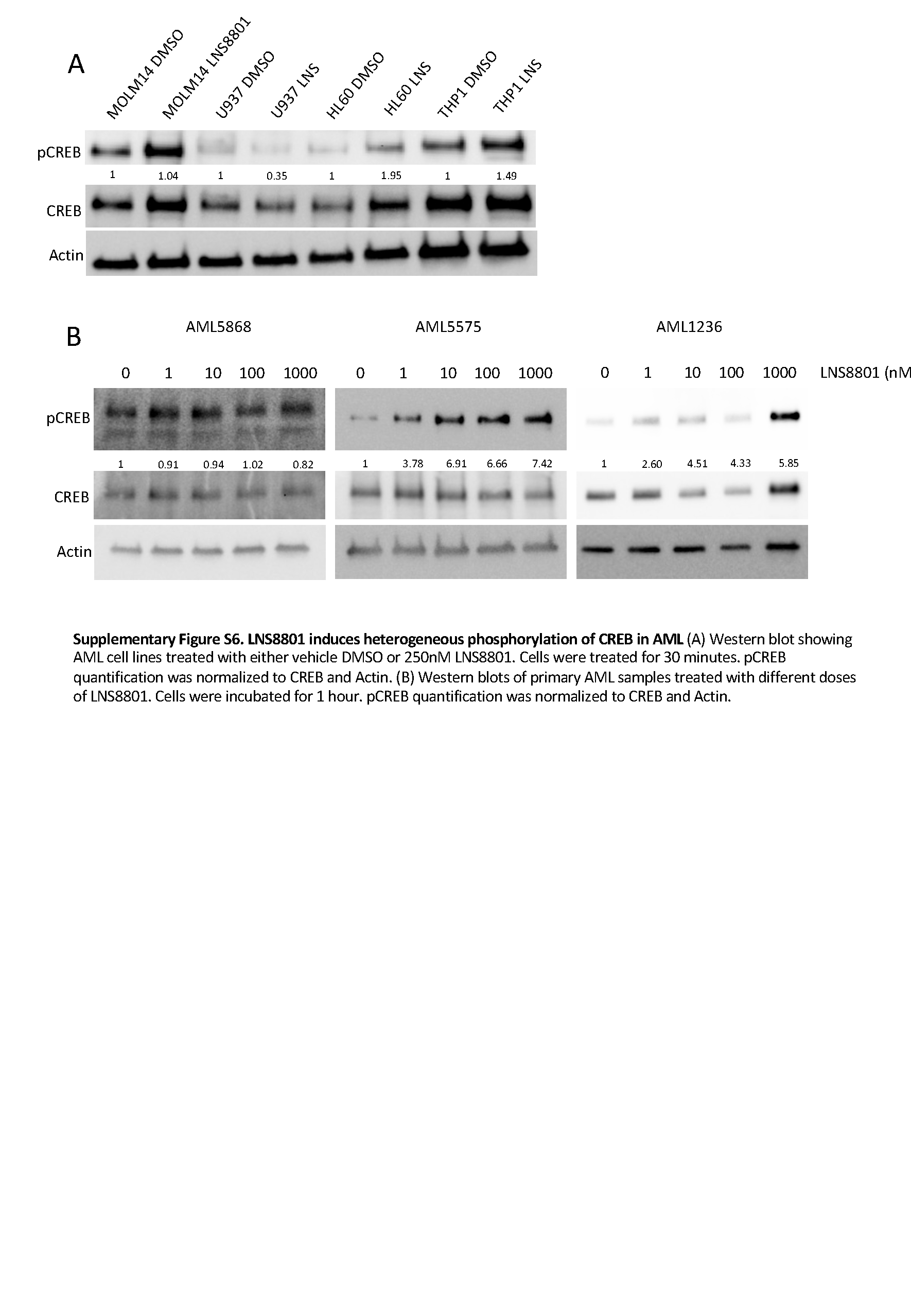

Supplement: Supplementary Figure S6 — LNS8801 induces heterogeneous phosphorylation of CREB in AML (A) Western blot showing AML cell lines treated with either vehicle DMSO or 250nM LNS8801. Cells were treated for 30 minutes. pCREB quantification was normalized to CREB and Actin. (B) Western blots of primary AML samples treated with different doses of LNS8801. Cells were incubated for 1 hour. pCREB quantification was normalized to CREB and Actin. [file crc-22-0478-s08.png]

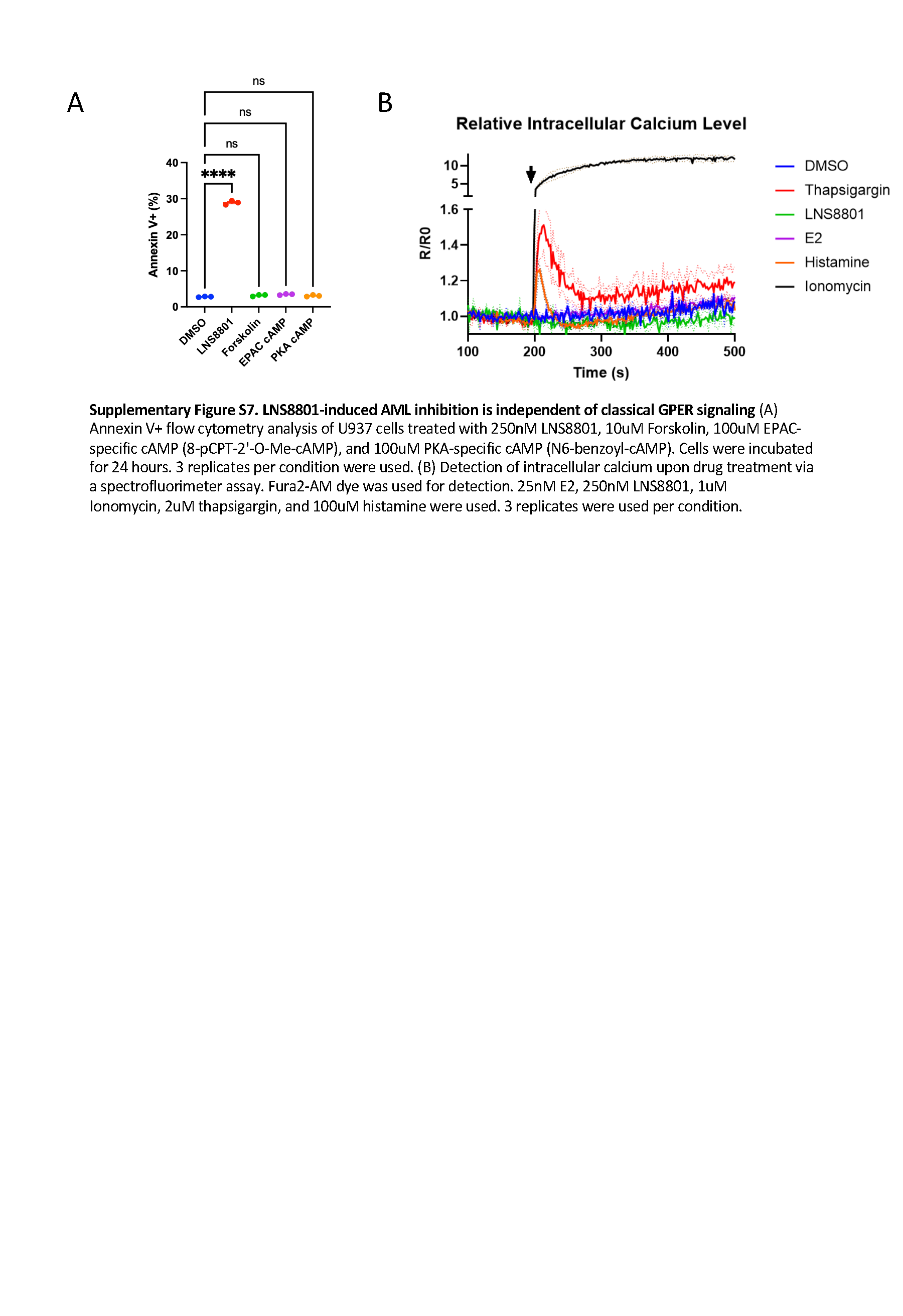

Supplement: Supplementary Figure S7 — LNS8801-induced AML inhibition is independent of classical GPER signaling (A) Annexin V+ flow cytometry analysis of U937 cells treated with 250nM LNS8801, 10uM Forskolin, 100uM EPAC-specific cAMP (8-pCPT-2'-O-Me-cAMP), and 100uM PKA-specific cAMP (N6-benzoyl-cAMP). Cells were incubated for 24 hours. 3 replicates per condition were used. (B) Detection of intracellular calcium upon drug treatment via a spectrofluorimeter assay. Fura2-AM dye was used for detection. 25nM E2, 250nM LNS8801, 1uM Ionomycin, 2uM thapsigargin, and 100uM histamine were used. 3 replicates were used per condition. [file crc-22-0478-s09.png]

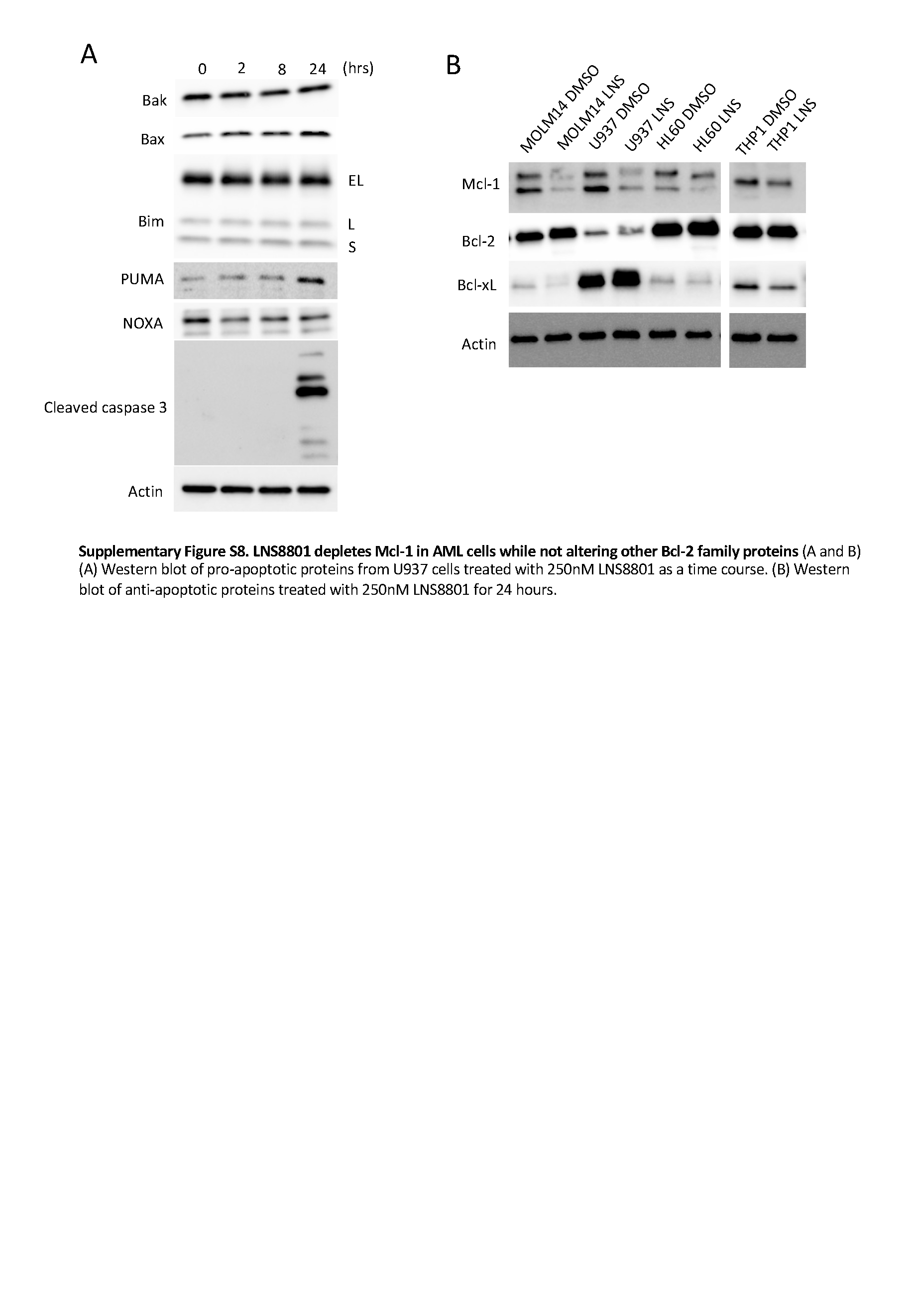

Supplement: Supplementary Figure S8 — LNS8801 depletes Mcl-1 in AML cells while not altering other Bcl-2 family proteins (A and B) (A) Western blot of pro-apoptotic proteins from U937 cells treated with 250nM LNS8801 as a time course. (B) Western blot of anti-apoptotic proteins treated with 250nM LNS8801 for 24 hours. [file crc-22-0478-s10.png]

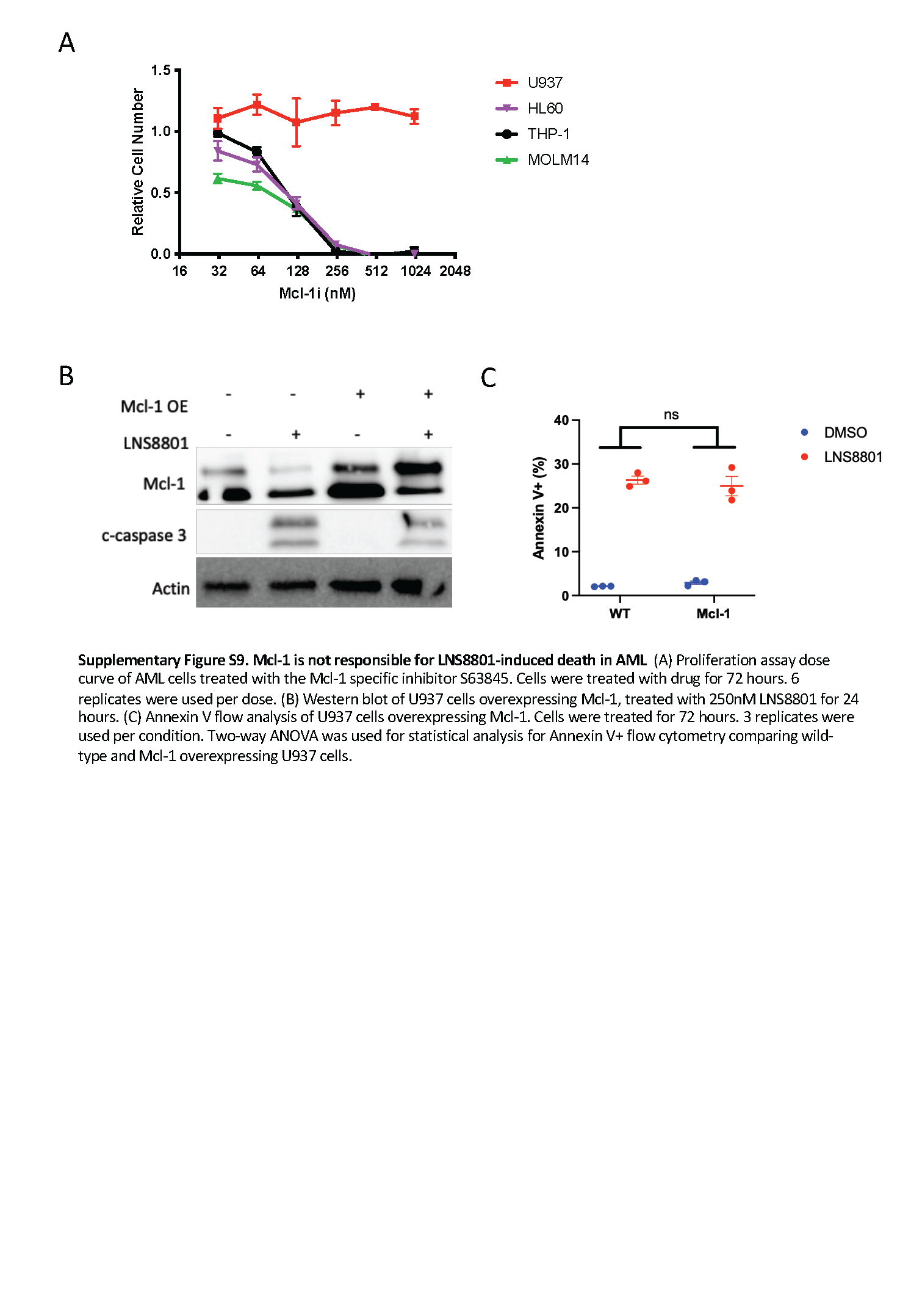

Supplement: Supplementary Figure S9 — Mcl-1 is not responsible for LNS8801-induced death in AML (A) Proliferation assay dose curve of AML cells treated with the Mcl-1 specific inhibitor S63845. Cells were treated with drug for 72 hours. 6 replicates were used per dose. (B) Western blot of U937 cells overexpressing Mcl-1, treated with 250nM LNS8801 for 24 hours. (C) Annexin V flow analysis of U937 cells overexpressing Mcl-1. Cells were treated for 72 hours. 3 replicates were used per condition. Two-way ANOVA was used for statistical analysis for Annexin V+ flow cytometry comparing wild-type and Mcl-1 overexpressing U937 cells. [file crc-22-0478-s11.png]

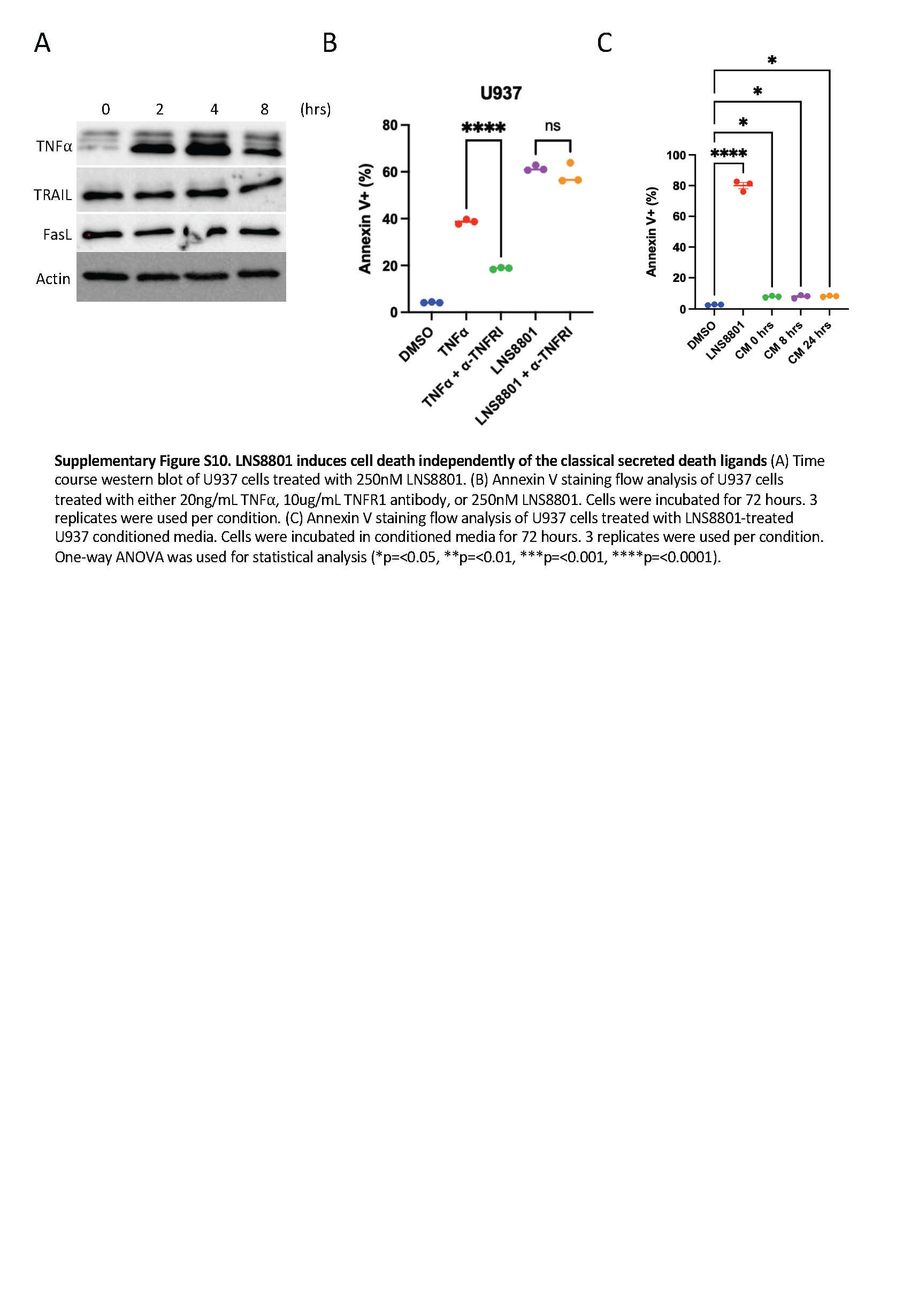

Supplement: Supplementary Figure S10 — LNS8801 induces cell death independently of the classical secreted death ligands (A) Time course western blot of U937 cells treated with 250nM LNS8801. (B) Annexin V staining flow analysis of U937 cells treated with either 20ng/mL TNF⍺, 10ug/mL TNFR1 antibody, or 250nM LNS8801. Cells were incubated for 72 hours. 3 replicates were used per condition. (C) Annexin V staining flow analysis of U937 cells treated with LNS8801-treated U937 conditioned media. Cells were incubated in conditioned media for 72 hours. 3 replicates were used per condition. One-way ANOVA was used for statistical analysis (*p=<0.05, **p=<0.01, ***p=<0.001, ****p=<0.0001). [file crc-22-0478-s12.png]

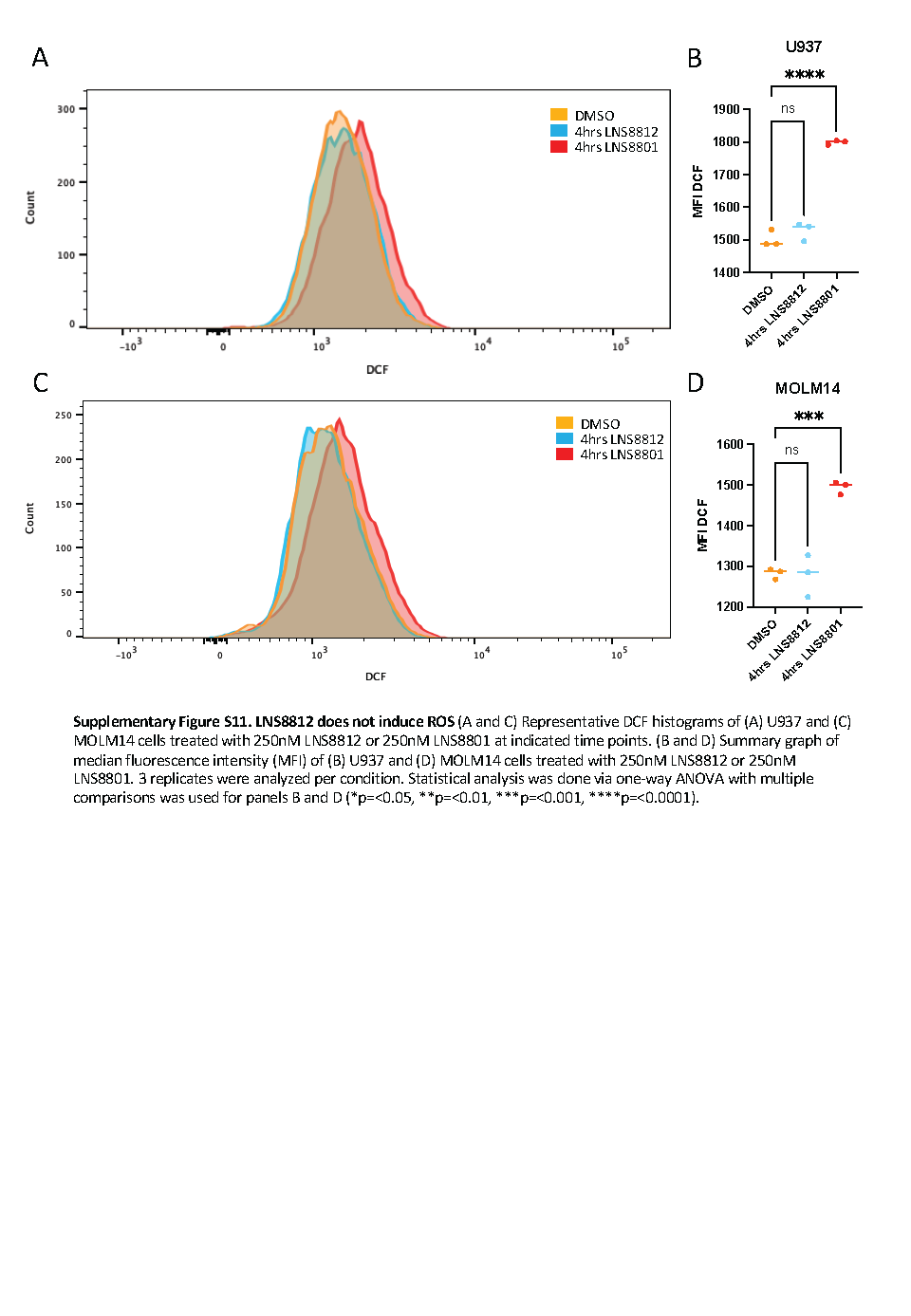

Supplement: Supplementary Figure S11 — (A and C) Representative DCF histograms of (A) U937 and (C) MOLM14 cells treated with 250nM LNS8812 or 250nM LNS8801 at indicated time points. (B and D) Summary graph of median fluorescence intensity (MFI) of (B) U937 and (D) MOLM14 cells treated with 250nM LNS8812 or 250nM LNS8801. 3 replicates were analyzed per condition. Statistical analysis was done via one-way ANOVA with multiple comparisons was used for panels B and D (*p=<0.05, **p=<0.01, ***p=<0.001, ****p=<0.0001). [file crc-22-0478-s13.png]

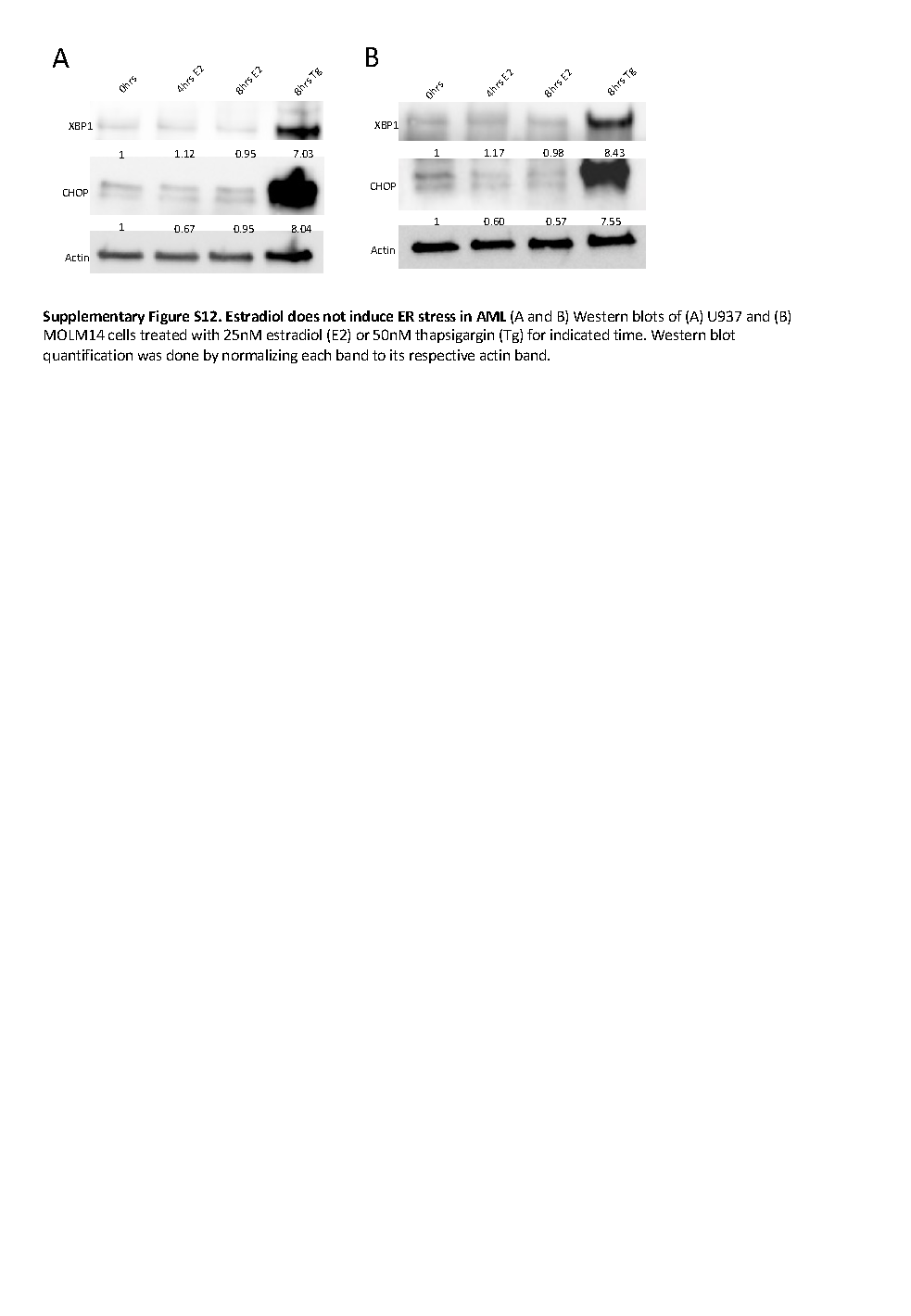

Supplement: Supplementary Figure S12 — (A and B) Western blots of (A) U937 and (B) MOLM14 cells treated with 25nM estradiol (E2) or 50nM thapsigargin (Tg) for indicated time. Western blot quantification was done by normalizing each band to its respective actin band. [file crc-22-0478-s14.png]

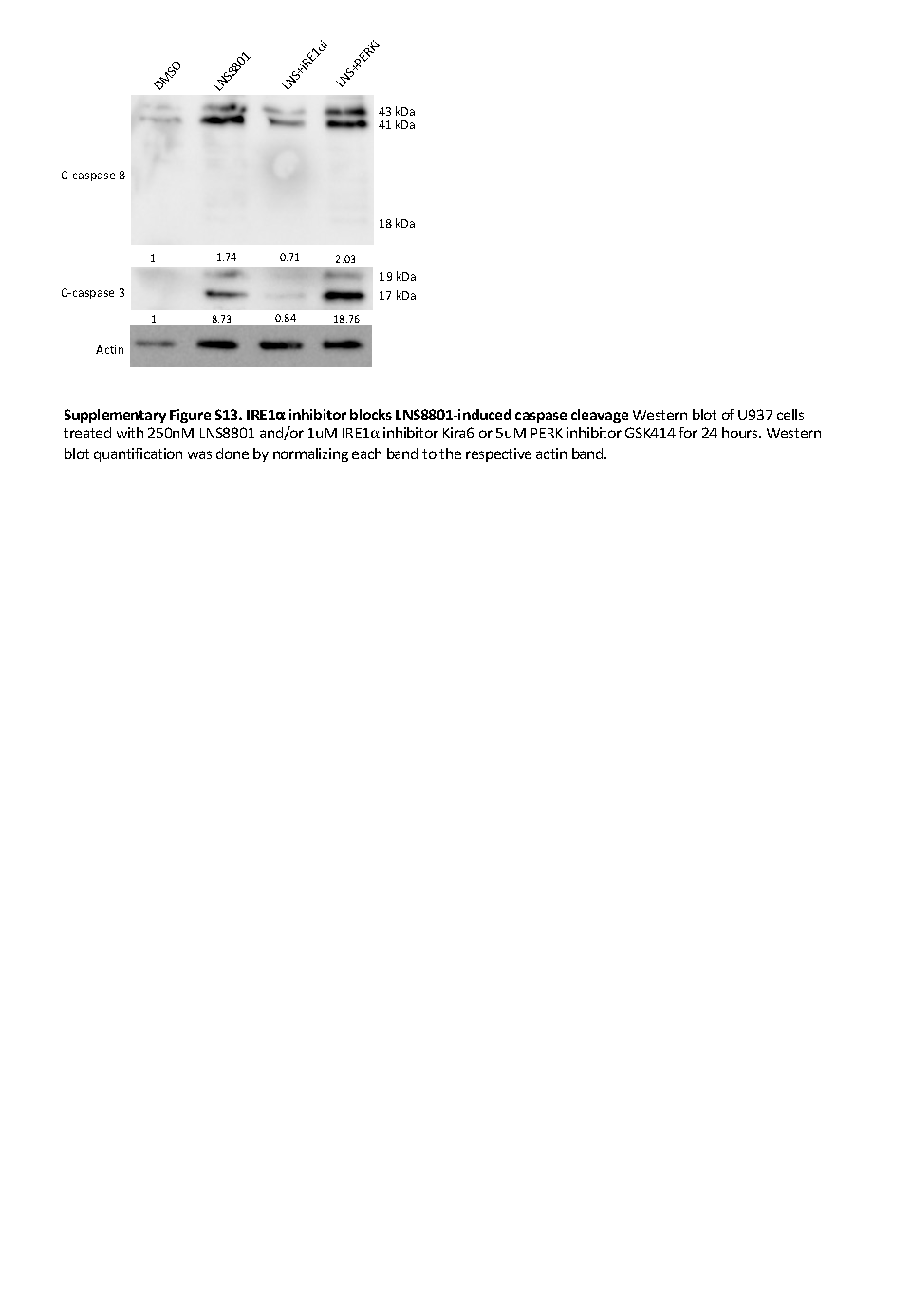

Supplement: Supplementary Figure S13 — IRE1⍺ inhibitor blocks LNS8801-induced caspase cleavage Western blot of U937 cells treated with 250nM LNS8801 and/or 1uM IRE1⍺ inhibitor Kira6 or 5uM PERK inhibitor GSK414 for 24 hours. Western blot quantification was done by normalizing each band to the respective actin band. [file crc-22-0478-s15.png]

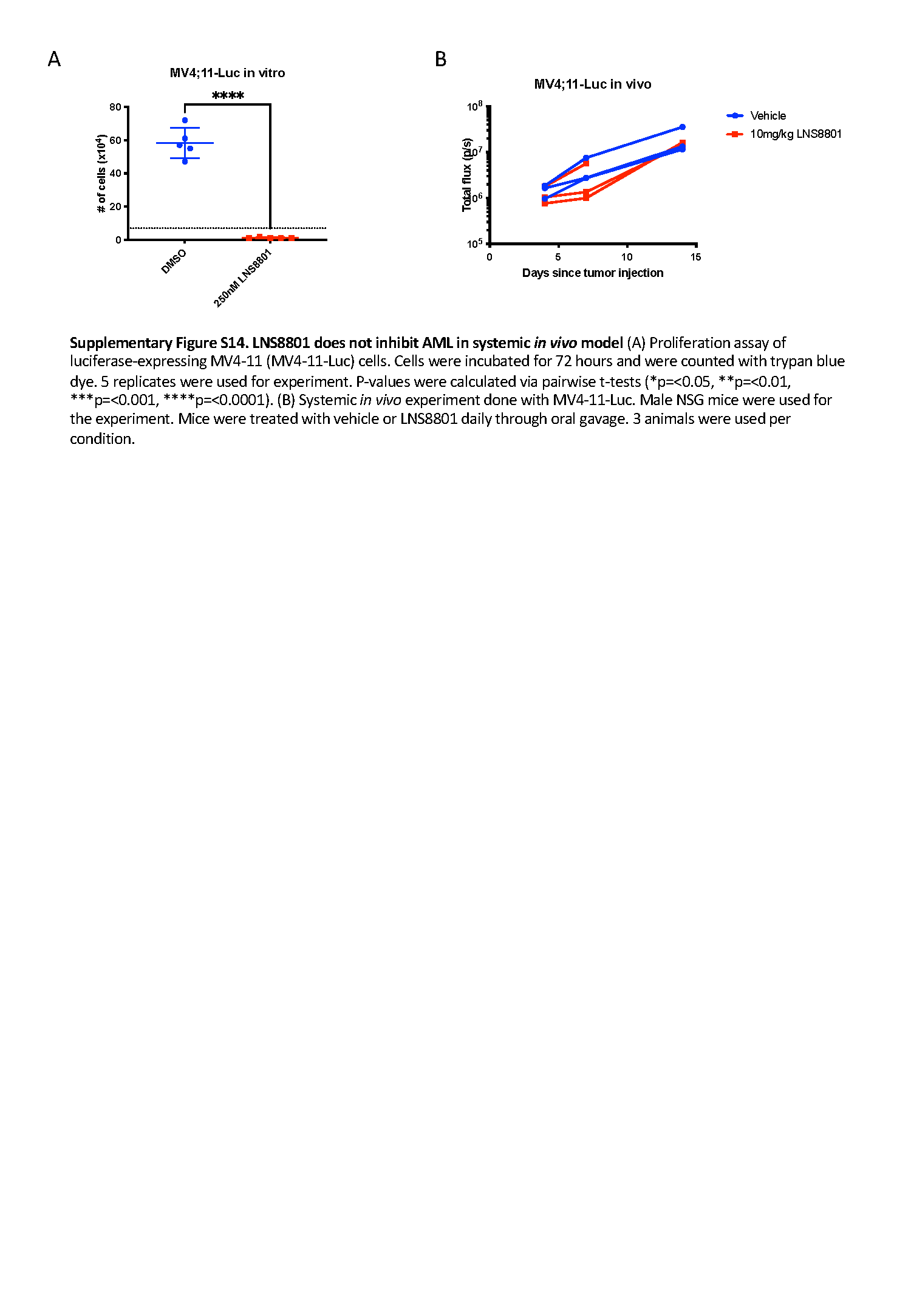

Supplement: Supplementary Figure S14 — LNS8801 does not inhibit AML in systemic in vivo model (A) Proliferation assay of luciferase-expressing MV4-11 (MV4-11-Luc) cells. Cells were incubated for 72 hours and were counted with trypan blue dye. 5 replicates were used for experiment. P-values were calculated via pairwise t-tests (*p=<0.05, **p=<0.01, ***p=<0.001, ****p=<0.0001). (B) Systemic in vivo experiment done with MV4-11-Luc. Male NSG mice were used for the experiment. Mice were treated with vehicle or LNS8801 daily through oral gavage. 3 animals were used per condition. [file crc-22-0478-s16.png]
