# Supplementary material for: LNS8801 inhibits Acute Myeloid Leukemia by Inducing the Production of Reactive Oxygen Species and Activating the Endoplasmic Reticulum Stress Pathway
Source: Cancer Res Commun. 2023 Aug 18;3(8):1594–606. doi: 10.1158/2767-9764.CRC-22-0478 (PMC10438922; doi:10.1158/2767-9764.CRC-22-0478)
Supplement: Supplementary Table S2 [file crc-22-0478-s03.pptx]

## Slide 1
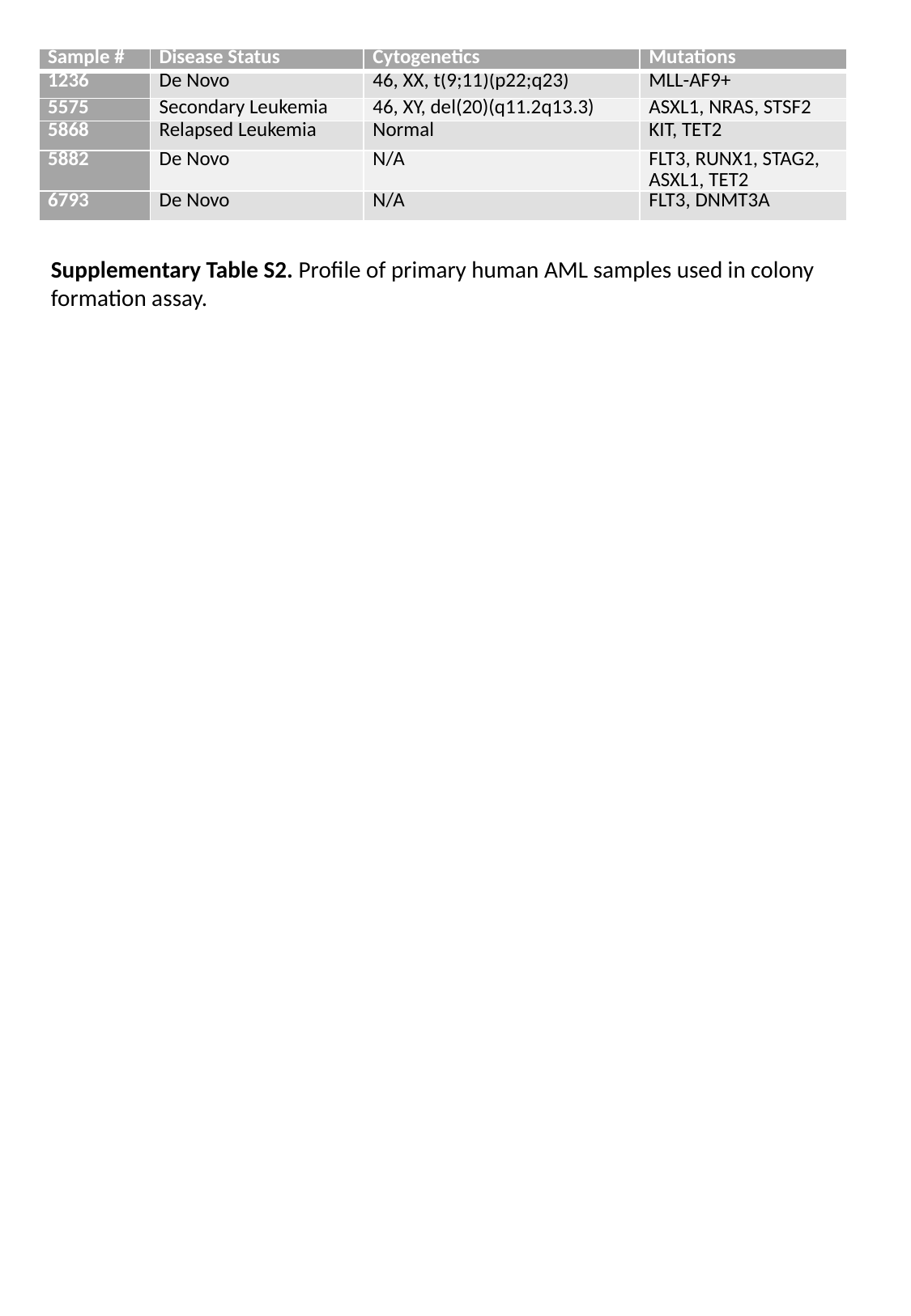

| Sample # | Disease Status | Cytogenetics | Mutations |
| --- | --- | --- | --- |
| 1236 | De Novo | 46, XX, t(9;11)(p22;q23) | MLL-AF9+ |
| 5575 | Secondary Leukemia | 46, XY, del(20)(q11.2q13.3) | ASXL1, NRAS, STSF2 |
| 5868 | Relapsed Leukemia | Normal | KIT, TET2 |
| 5882 | De Novo | N/A | FLT3, RUNX1, STAG2, ASXL1, TET2 |
| 6793 | De Novo | N/A | FLT3, DNMT3A |
Supplementary Table S2. Profile of primary human AML samples used in colony formation assay.
